# Reconstructing Evolutionary Histories with Hierarchical Orthologous Groups

**DOI:** 10.1007/s00239-025-10277-1

**Published:** 2025-11-21

**Authors:** Garance Sarton-Lohéac, Nikolai Romashchenko, Clément Marie Train, Sina Majidian, Natasha Glover

**Affiliations:** 1 Department of Fundamental Microbiology, University of Lausanne, 1015 Lausanne, Switzerland; 2 Department of Computational Biology, University of Lausanne, 1015 Lausanne, Switzerland; 3 SIB Swiss Institute of Bioinformatics, 1015 Lausanne, Switzerland; 4 Department of Computer Science, Johns Hopkins University, 3400, North Charles St., Baltimore, MD 21218 USA

**Keywords:** Hierarchical orthologous groups, Orthology, Paralogy, Comparative genomics, Ancestral genomes, Phylogenetics, Orthology benchmarking, Gene function

## Abstract

With the rapid advancement of large-scale sequencing initiatives, the need for efficient and accurate methods for inferring orthologous and paralogous relationships has never been more critical. Hierarchical orthologous groups (HOGs) provide a powerful solution to this challenge, offering a precise, scalable framework to study gene families and their evolutionary histories across diverse species. In this review, we introduce the concept of HOGs and explore their advantages over traditional methods. Next, we highlight key applications of HOGs, including their use in representing gene families, inferring ancestral genomes, tracking gene gain and loss events, functional annotation, and phylogenetic profiling. We overview the process of constructing HOGs and discuss the challenges and limitations of HOG inference. The HOG framework provides a clear and structured approach to organizing homologous genes, making it possible to gain deeper insights into gene family and species evolution.

## Introduction

The explosion of sequencing data from projects such as the Earth Biogenome Project and the European Reference Genome Atlas has revolutionized comparative genomics (Lewin et al. 2022; Mazzoni et al. 2023). Access to genomic data across diverse species improves our ability to study gene function and evolution, which is critical for understanding the molecular basis of biological processes. Genes related by shared ancestry are called homologs, further classified as orthologs or paralogs based on their evolutionary origin (Fitch 1970). Orthologs are genes that arose from a speciation event; in contrast, paralogs originate from a gene duplication event, leading to the parallel descent of two or more copies within a lineage (Box 1). The concept of orthology has been thoroughly reviewed in other papers, e.g., (Altenhoff et al. 2019; Fernández et al. 2020; Koonin 2005), and distinguishing orthologs from paralogs is essential for understanding gene function, evolution, and species relationships (Koonin 2005).

Orthology is inherently hierarchical: genes generally evolve via vertical descent, forming a nested structure of shared ancestry at different taxonomic “levels of orthology” (van der Heijden et al. 2007). Orthologous groups can thus be defined at multiple evolutionary depths. However, analyzing multiple species or large, duplication-rich gene families is challenging due to potential inaccuracies in gene trees and thus difficulties in inferring orthologs and paralogs with gene tree–species tree reconciliation. Traditional pairwise orthology inference methods identify one-to-one orthologous pairs of genes or one-to-many relationships in flat orthologous groups at a single level, but often fail to capture when duplication events occurred, making it difficult to distinguish orthologs from in-paralogs. Without a hierarchical framework, homologs form an undifferentiated “bag of genes,” obscuring whether duplications predate or postdate a speciation event (Fig. 3). These challenges highlight the need for more nuanced frameworks, such as hierarchical orthologous groups (HOGs) (Jothi et al. 2006; van der Heijden et al. 2007), which represent gene evolution across multiple taxonomic levels in a phylogenetic context.

In this review, we begin with defining the concept of HOGs and describe its applications, including determining gene family evolutionary history, gene family emergence, expansions and contractions, ancestral genome reconstruction, ancestral gene order, functional annotation, and phylogenetic profiling. We then review methods for constructing HOGs and discuss current limitations. By outlining both strengths and challenges, we aim to provide a balanced view of how HOGs can be integrated into comparative genomics workflows.

**Fig. 1:**
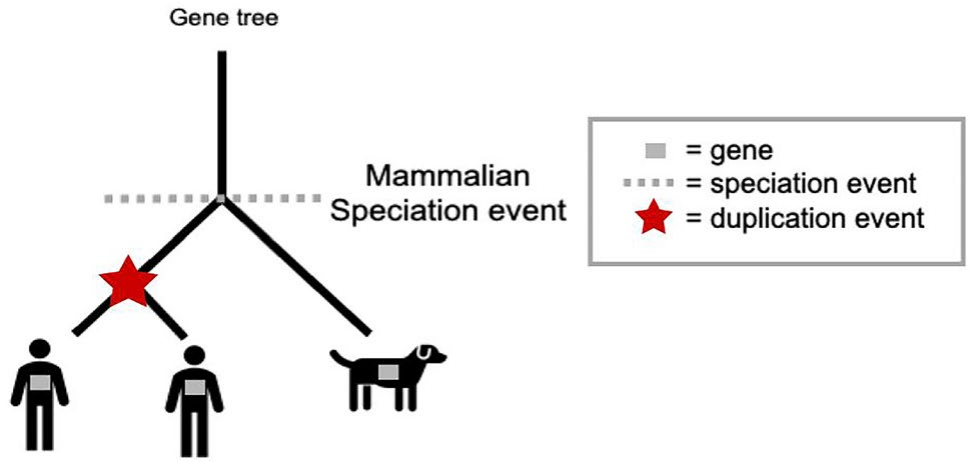
A homologous gene family can be represented by a gene tree. Here, a duplication event resulted in two copies of the gene in human, yet one in dog

Box 1. Homology: key concepts Homology refers to genes that descended from a common ancestral gene. A gene tree depicts the evolutionary relationships among various homologous genes, i.e., modern-day genes which derived from an ancestral gene. The process of reconciliation compares the gene tree to the species tree to annotate each internal node of the gene tree as a speciation or duplication event. All gene pairs in Figure 1 are homologous and form a gene family.

**Orthology vs. paralogy**

Homology, and its subtypes orthology and paralogy, each represent *relations* between pairs of genes. The evolutionary event which caused the two genes to start diverging distinguishes the relationship:

- Orthology: A relation between pairs of genes that started diverging via speciation.
- Paralogy: A relation between pairs of genes that started diverging via duplication.

The *pairs of genes* themselves are referred to as orthologs or paralogs:

- Orthologs: pairs of genes that started diverging via speciation.
- Paralogs: pairs of genes that started diverging via duplication.

To determine if the point of divergence between two genes was a duplication or speciation, one can trace back up the reconciled gene tree to the point where the two genes coalesce.

**In-paralogy vs. out-paralogy**

In-paralogy and out-paralogy are relationships defined over a pair of genes with respect to a reference speciation.

- In-paralogs: genes duplicated *after* the speciation event of reference.
- Out-paralogs: genes duplicated *before* the speciation event of reference.

**Fig. 2:**
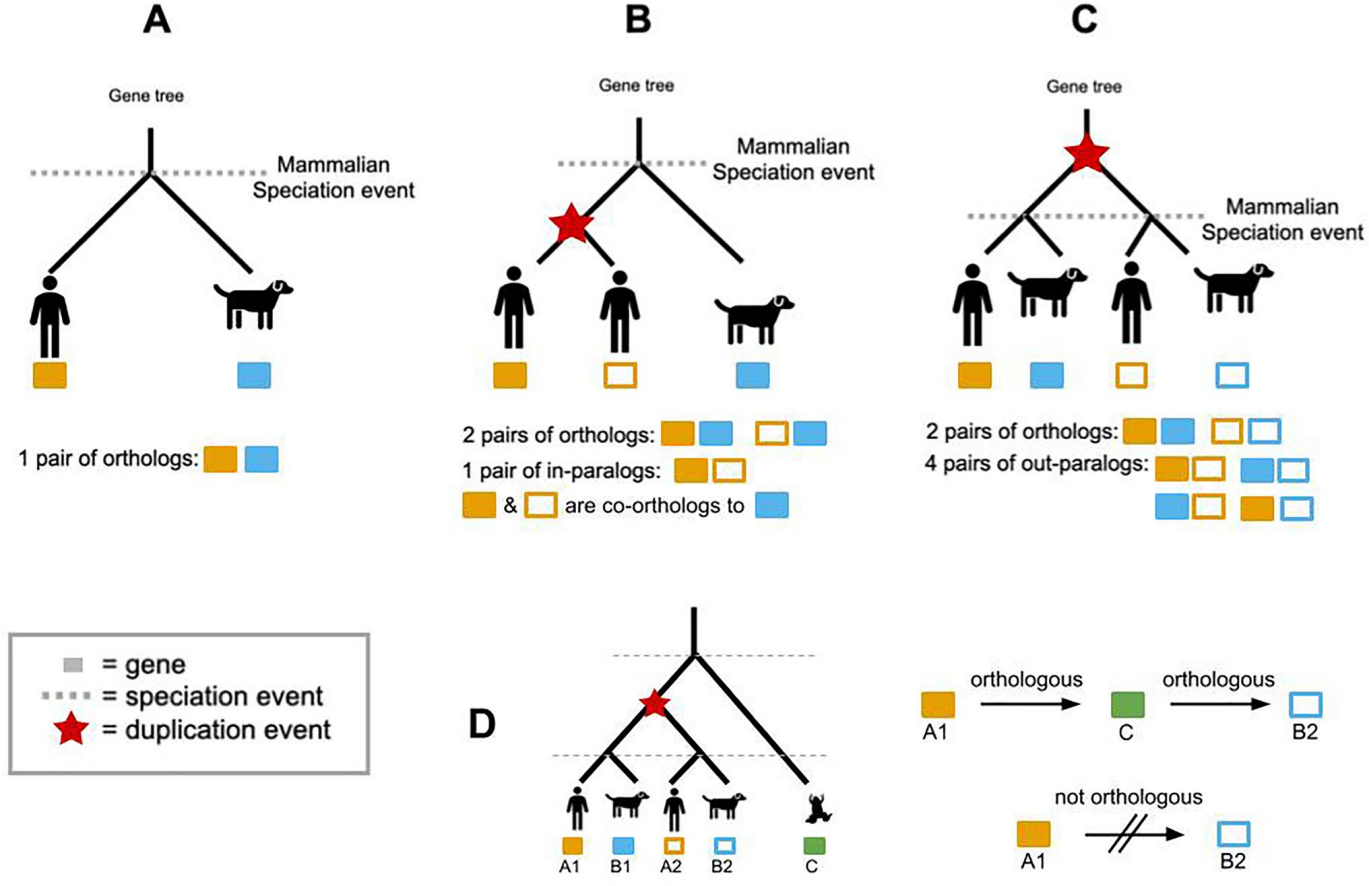
Illustration of in-paralogs, out-paralogs, and co-orthologs. **A** A simple evolutionary scenario where the divergence of dog and human started at the mammalian taxonomic level. A gene which was one gene in the mammals common ancestor is now two orthologous genes, one in each species. **B** Scenario where a gene duplication happened in the human lineage. Now the two copies of the human gene are co-orthologs to the one dog gene. The two human genes are also in-paralogs with respect to the mammals speciation. **C** Scenario where the duplication event occurred in the common ancestor of humans and dogs, before the mammalian speciation. There are now two pairs of orthologs and four pairs of out-paralogs. **D** Example of the non-transitive property between a triplet of co-orthologs. Genes A1 and B2 are orthologous to outgroup C, but A1 and B2 are paralogous

The relative timing of the duplication and speciation events affects the induced pairs of orthologs and paralogs. In Figure 2b, there is one pair of in-paralogs because the duplication event occurred after the reference speciation. However, if the duplication occurred before the reference speciation (Figure 2c), there are now 4 pairs of out-paralogs. It is important to note that paralogs may exist in different species, and their presence is solely dependent on their evolutionary origin (duplication).

**Co-orthology**

Co-orthology is a relationship defined over three or more genes. In the case of a lineage-specific duplication, the resulting duplicates are both co-orthologous to the outgroup gene.

Orthology is a relationship between pairs of genes. However, pairwise orthologous relationships are non-transitive, meaning a gene can be orthologous to two other genes but the latter may not be orthologous to each other due to duplications before speciation events of reference (Figure 2d). These paralogs are both orthologous to an outgroup gene, but not to each other. This motivates the need for methods which guarantee non-transitivity of phylogenetic relationships, so as to not mistakenly identify paralogs for orthologs.

## Definition of HOGs

Throughout this review, we use “genome” to refer to the protein-coding content of a species (i.e., its proteome), which is the focus of most orthology inference methods. Similarly, we use “gene” and “protein” interchangeably to refer to protein-coding genes.

In this section, we introduce the conceptual basis of HOGs and explain how they represent gene evolution at different taxonomic levels.

### The HOG Framework

The HOG framework is a holistic approach to comparative genomics that reconstructs the evolutionary histories of all gene families in the multiple species under consideration. Unlike traditional approaches that consider genes at a single evolutionary level, the HOG framework systematically organizes homologous genes across multiple taxonomic depths, using the species phylogeny as a guide. HOGs can be inferred from phylogenetic gene trees, graph-based clustering, or with hybrid methods that combine elements of both. Beyond identifying orthologous relationships, HOGs capture duplications, losses, and ancestral gene content, in a structured and taxonomically informed manner. This framework is especially suitable for large-scale analyses, as resulting orthology and estimations for ancestral gene content are readily accessible for any taxonomic level without additional computations. By embedding gene families within a hierarchical structure, the HOG framework unifies multiple perspectives on gene evolutionary relationships into a single, coherent evolutionary model.

**Fig. 3:**
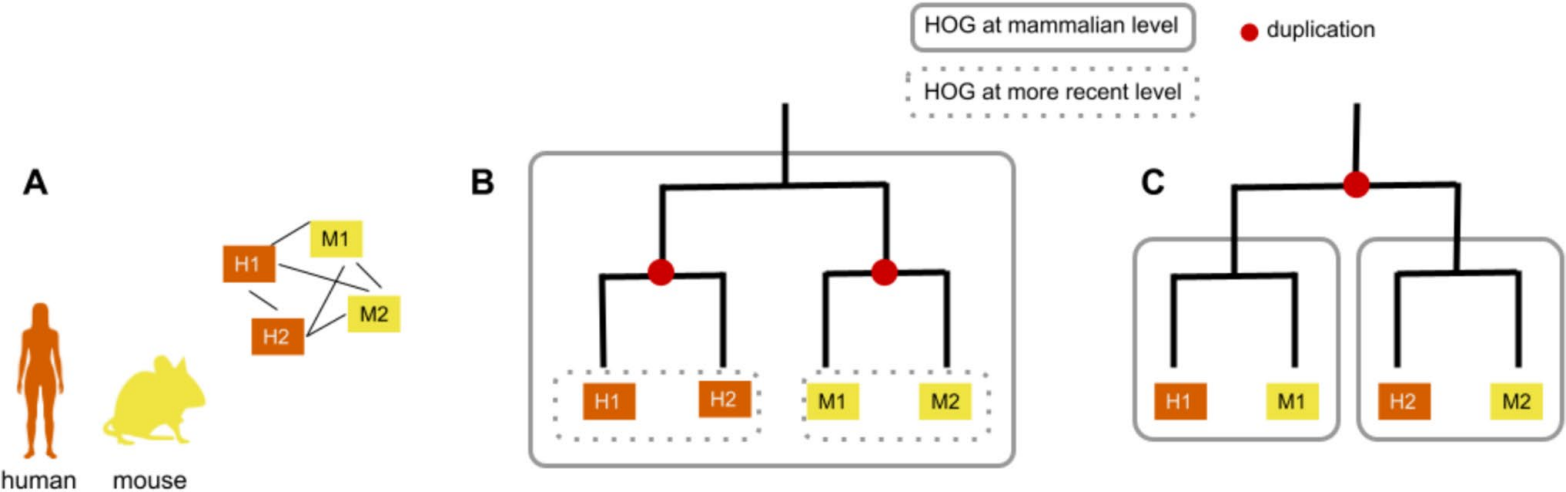
HOGs make it possible to deduce when duplication events occurred. **A** A flat group of homologous genes without a gene tree or HOG. With the absence of other information, the homologs are a “bag of genes.” One cannot know if the duplication event which gave rise to the two copies in each species happened before or after the last common ancestor (LCA). **B** Scenario 1, where two independent duplications occurred, one in the human lineage and one in the mouse lineage. **C** Scenario 2, where an ancestral duplication occurred in the LCA (e.g., Mammalia), before the divergence of mouse and human. With the HOG framework, one HOG would be inferred at the mammalian level in scenario 1, and two HOGs would be inferred at the mammalian level in scenario 2

### What HOGs Represent

A HOG represents a set of genes descended from a single ancestral gene, defined with respect to a given taxonomic level. There are several equivalent ways to conceptualize a HOG, each offering a complementary perspective:

#### Groups of Extant Orthologs and Paralogs, Defined with Respect to a Taxonomic Level

**Fig. 4:**
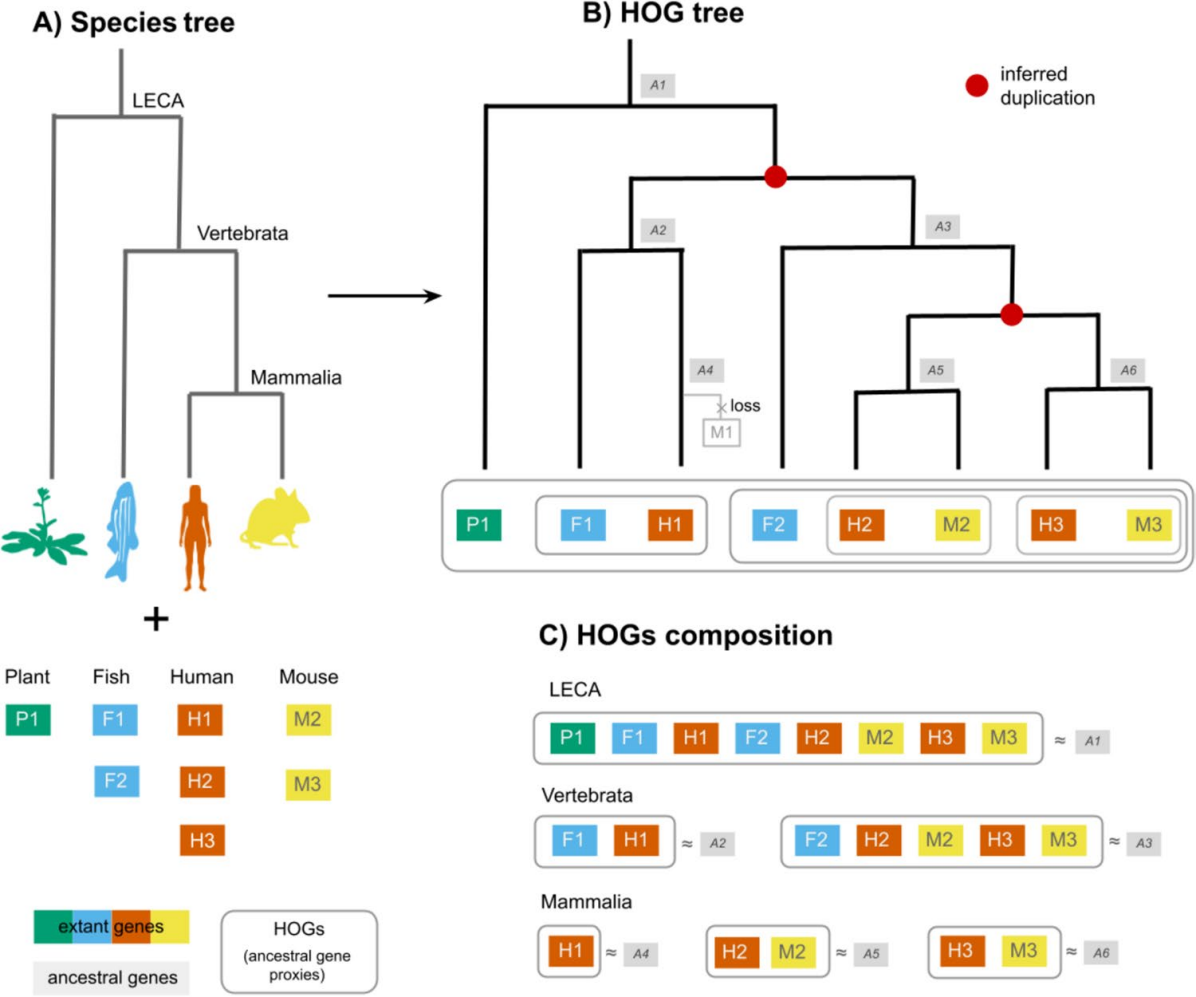
Conceptual overview of hierarchical orthologous groups. An example of one HOG, or gene family. **A** Species tree with four taxa: plant (green), fish (blue), human (orange), and mouse (yellow), each with one or more genes. **B** The implied gene tree, dubbed “HOG tree,” and inferred nested HOG composition. Duplication nodes (red) can be deduced based on the species tree topology and clusters of homologous genes at each level. Ancestral genes from which the HOGs descended are shown in gray. **C** HOGs returned at different taxonomic levels. Consider a gene family that was present in the last eukaryotic common ancestor (LECA). At this level, a single HOG encompasses all genes descending from that ancestral gene. At the Vertebrata level, this gene underwent duplication, leading to two distinct copies, i.e., HOGs. At the Mammalia level, a second duplication further subdivides one of these HOGs, showing how deeper HOGs split into nested subHOGs at more recent levels. The HOG composition implies that a loss event occurred after the mammalian speciation

A HOG is a set of homologous genes found in extant species that have all descended from a single ancestral gene at a specified taxonomic level, i.e., at every internal node in the species tree (Fig. 4). For example, a HOG defined at the level of the last eukaryotic common ancestor (LECA) contains all genes derived from a single LECA gene, including post-LECA duplications. At a more recent level (e.g., Vertebrata), we would define a new set of HOGs based on the genes present in the last common ancestor of vertebrates, potentially dividing that broader LECA-level HOG into multiple HOGs due to gene duplication, or removal of HOGs due to gene loss. Barring inference errors, HOGs allow users to select the level of granularity in a precisely defined and evolutionarily meaningful way, reflecting gene descent from a common ancestral copy (Sonnhammer et al. 2014).

#### Clades on Reconciled Gene Trees

**Fig. 5:**
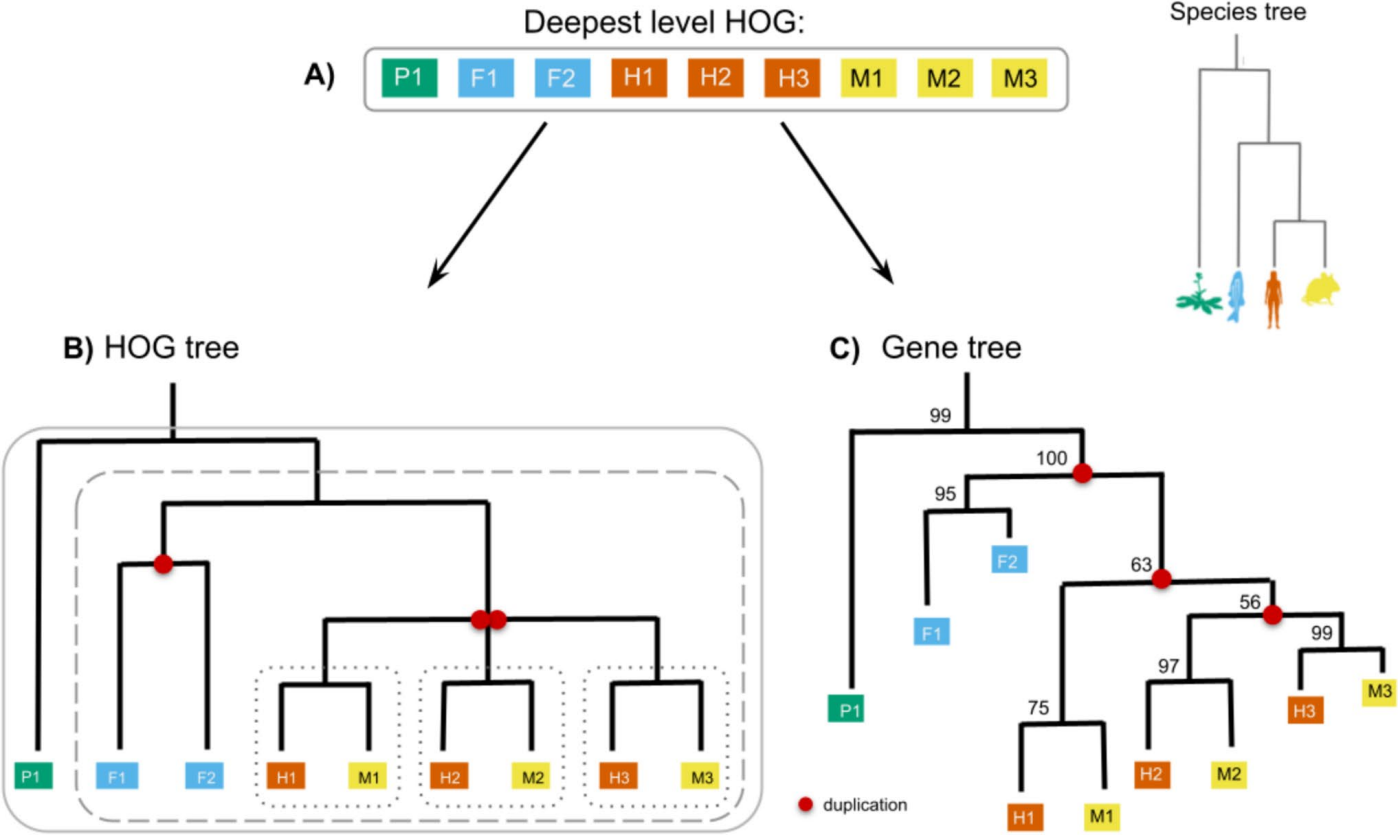
The relationship between HOGs and gene trees. **A** A HOG is a set of extant genes (homologs) descended from a common ancestral gene. **B** The corresponding HOG tree, i.e., a cladogram showing speciation and duplication events but lacks branch length information. **C** A fully reconstructed gene tree, inferred using methods such as maximum likelihood, which includes branch lengths and bootstrap values. Such trees can clarify the order of duplications but may be harder to interpret or compute for large families

From a phylogenetic perspective, HOGs can be considered as clades within a reconciled gene tree (Fig. 5). In reconciled trees, internal nodes are labeled as either speciation or duplication events (Menet et al. 2022; Page & Charleston 1997). A HOG at a given taxonomic level corresponds to the clade rooted at the speciation node representing the last common ancestor of the genes in that clade. For instance, a HOG at the Mammalia level corresponds to a clade of genes in a reconciled tree where all the extant species are mammals. Gene duplications occurring before this node define separate HOGs (clades) at this level, while duplications occurring afterward form subclades within the HOG. A one-to-one correspondence exists between HOGs and labeled gene trees. Both contain information about speciation and duplication events, but they are encoded in different data structures: HOGs as nested sets of genes, gene trees as bifurcating tree structures with additional information such as branch lengths (discussed further in the Gene Family Evolutionary History section).

#### Gene Families and Subfamilies

HOGs provide a structured way to define gene families and subfamilies. At the deepest taxonomic level, a HOG represents an entire gene family—the set of all genes descending from a single common ancestor. As one moves toward more recent species, the family is progressively subdivided into smaller, nested groups, corresponding to gene subfamilies. This hierarchical organization aligns well with common practices in molecular evolution and functional genomics, which allows for fine-grained analysis of gene function and adaptation (discussed further in the Functional Annotation section).

#### Ancestral Gene Proxies

At any given taxonomic level, each HOG represents a set of extant genes that descended from a single ancestral gene (Fig. 4). In this sense, the HOG itself serves as a proxy for that ancestral gene. The nested structure of HOGs allows us to trace these ancestral genes across evolutionary time, with duplications and losses annotated explicitly. When considered together, the full set of HOGs at a particular level approximates the ancestral genome: each HOG corresponds to one ancestral gene, and their union represents the gene repertoire of the common ancestor. This perspective makes HOGs a natural framework for ancestral genome reconstruction, even when that goal is not explicitly stated (discussed further in the Ancestral Genome Reconstruction section).

The concept of HOGs was introduced in 2006 by Jothi et al., who proposed integrating species phylogeny into ortholog clustering (Jothi et al. 2006). Their algorithm, COCO-CL, inferred hierarchical groups by progressively clustering genes along the species tree, allowing for orthology analysis at multiple taxonomic levels. Since then, HOGs have been widely adopted, with numerous computational tools and databases that implement hierarchical orthology inference, including LOFT (van der Heijden et al. 2007), eggNOG (Jensen et al. 2008), OrthoDB (Kriventseva et al. 2008), PhylomeDB (Huerta-Cepas et al. 2008), EnsemblCompara (Vilella et al. 2009), PANTHER (Mi et al. 2010), MBGD (Uchiyama et al. 2013), OMA (Altenhoff et al. 2013), Hieranoid (Schreiber & Sonnhammer 2013), and OrthoFinder (Emms & Kelly 2015).

## Applications of Hierarchical Orthologous Groups

Understanding gene evolutionary history is central to comparative genomics. Traditional orthology methods have provided valuable insights, but large-scale analyses often require *ad hoc* reconciliation of orthologs and paralogs across multiple genomes. HOGs streamline this process by providing a structured framework to integrate gene family evolution with species phylogeny, making sure that orthologs and paralogs are assigned in a biologically meaningful way. The next section highlights key applications of HOGs.

### Gene Family Evolutionary History

One of the most direct uses of HOGs is tracing the evolutionary history of a gene family. At its deepest level, each HOG represents a gene family, encompassing all homologs descended from a single ancestral gene. The hierarchical structure enables reconstruction of a gene family’s full evolutionary trajectory across multiple taxonomic depths, i.e., inferring where in the species’ phylogeny duplications or losses occurred.

While HOGs capture the order of duplications and speciations, further phylogenetic analyses such as gene tree reconstruction methods like maximum likelihood can refine evolutionary histories by estimating branch lengths, providing statistical support with models of sequence evolution or bootstrapping, and resolving the order of duplications between speciation events if there are multiple such duplications (Fig. 5). However, gene trees can be cumbersome to interpret for large, duplication-rich families. The challenge for the most extreme cases of duplication-rich gene families (e.g., olfactory receptors in animals, MYB transcription factors in plants) is that the inevitable inaccuracies of their gene trees make it difficult to infer the duplication timing by reconciling the gene tree with the species tree. Gene tree inference can also be computationally intensive, depending on the scale and method used. Nevertheless, several HOG methods infer gene trees to benefit from them in various ways, despite these issues.

The hierarchical framework of HOGs is especially useful where large numbers of gene duplications occurred due to whole genome duplication (e.g., in plants), as whole genome duplication produces many duplications in a single event and may therefore be easier to detect. This idea was exemplified by the PhyloMCL software, a HOG inference method used to detect polyploidy events in plant genomes, where candidate whole genome duplications are attributed to nodes on the species tree if the HOG numbers were four times higher than those of the parent or child nodes (Zhou et al. 2020). This idea was also used by Moix et al. (2023), where candidate WGD events were determined in yeasts based on a significant increase in the number of duplicated gene families.

### Ancestral Genome Reconstruction

Reconstructing ancestral genomes is a key goal in comparative genomics, as it allows one to infer the gene content of extinct ancestors and trace genome evolution over time. Ancestral genome reconstruction has been a long-standing topic and may refer to the reconstruction of ancestral gene repertoires, DNA sequences, gene orders, and karyotypes (Rascol et al. 2007). In the context of this review, we refer to an ancestral genome as the estimated set of genes that existed in the last common ancestor of a given clade. This perspective has long been implicit in hierarchical orthology inference. For example, Schreiber and Sonnhammer introduced the term "pseudo-species" to refer to internal nodes in a species tree associated with orthologous groups at different taxonomic levels (2013). Later, Kaduk and Sonnhammer (2017) termed these "meta-species," emphasizing that HOG-based methods inherently reconstruct ancestral genomes, even if it is not explicitly framed as such.

Several resources now provide formal ancestral genome reconstructions based on HOGs. The Ancestral Genomes database (Huang et al. 2018), for instance, combines curated gene trees from the PANTHER database with parsimony-based inference of gene loss to estimate the gene content of ancestral species (Thomas 2010). There are currently 132 extant species, 111 ancestral species, and 1.2 million ancestral genes released by Ancestral Genomes (last accessed 17 March 2025). Each inferred ancestral gene is assigned a stable identifier, a predicted name, a reconstructed protein sequence, inferred Gene Ontology (GO) annotations, and a representative extant gene that serves as a proxy ancestral gene, chosen based on minimal divergence from the ancestral sequence.

Similarly, the OMA database uses the GETHOGs algorithm to infer HOGs across nearly 3000 extant genomes and 1133 ancestral genomes (Altenhoff et al. 2013; Altenhoff et al. 2024a, b). On the explicit ancestral genomes webpages, users can browse through the "ancestral genes" table, i.e., HOGs inferred at a specific taxonomic level. Users can explore gene gains, losses, and duplications in ancestral lineages.

Understanding the biology of key ancestral species at critical landmarks in evolution, such as LECA, will help us better understand their traits’ evolution. To do that, HOG inference is a crucial step (Richards et al. 2024). Ancestral genome reconstructions open the door to a wide range of applications, discussed in the following sections.

### Gene Family Emergence

A major strength of hierarchical orthology is its ability to estimate the “birth” of each gene family by mapping it to the deepest node where it is inferred to have existed—a concept known as phylostratigraphy. Originally proposed by Domazet-Lošo et al.(2007), the phylostratigraphy approach assigns each extant gene an evolutionary age based on the most ancestral node where an ortholog is found. In a HOG framework, this is straightforward: the deepest HOG containing a gene indicates the taxonomic level at which its ancestral copy arose.

HOG-based phylostratigraphy enables large-scale gene age profiling. For example, de Paiva Lopes et al. (2016) assigned all ~ 18,500 human protein-coding genes to HOGs across the tree of life to identify the lowest common ancestor for each gene. From this, the authors found eight major evolutionary transitions in terms of emergence of new genes. There was a burst of housekeeping genes emerging early in evolution, between LUCA and LECA. On the other hand, genes with tissue-specific expression tended to have emerged much more recently (around Mammalia) (de Paiva Lopes et al. 2016).

Paps and Holland (2018) used a HOG-like approach to infer the metazoan ancestral gene repertoire across 62 eukaryotic genomes. Although their method grouped all orthologs and paralogs from duplications that may have occurred before Metazoa (not a true HOG framework), they still identified over 6000 ancestral genes and revealed a burst of genomic novelty at the origin of animals. Notably, they pinpointed 25 metazoan-specific gene families conserved across all animal phyla but absent from unicellular relatives, suggesting these families were pivotal for the emergence of multicellularity.

Phylostratigraphy has also been applied at scale. For example, Domazet-Lošo et al. (2024) analyzed 667 genomes and found that the number of gene families peaks at key evolutionary transitions, such as the origins of eukaryotes and animals, and then declines toward extant lineages. Their findings suggest that bursts of innovation are often episodic, tied to evolutionary milestones, and are followed by periods of gene loss and refinement.

Such examples show how HOG-based phylostratigraphy allows researchers to identify key innovation points in evolution and characterize lineage-specific novelty.

### Gene Family Expansions and Contractions

Beyond gene emergence, HOGs enable systematic tracking of gene family expansion and contraction over time. By comparing HOG composition across taxonomic levels, researchers can identify where duplications or losses occurred and quantify their impact on gene family size.

One early study by Lespinet et al. (2002) identified lineage-specific expansions across five eukaryotic genomes. Even without the full HOG framework available at the time, they identified gene families that had expanded after the divergence of major taxa. The results showed a large fraction of genes belonging to lineage-specific expansions. For instance, up to ~ 80% of Arabidopsis proteins were in plant-specific expansions. These expanded families were enriched in functions such as stress response, immunity, and development, suggesting adaptive roles. More recent studies have confirmed these insights, e.g., (Gao et al. 2023; Kim et al. 2022; Stanley et al. 2022). Modern HOG methods theoretically allow precise localization of duplication and loss events on the species tree and comparison of expansion rates across clades.

In a phylogenomics study by Breeschoten et al. (2022), the authors applied the HOG framework to investigate gene family evolution across four focal Lepidoptera taxa. They used OrthoFinder to delineate HOGs and CAFE (De Bie et al. 2006) to estimate duplication rates across the phylogeny for gene families involved in plant-feeding adaptations. The authors found that gene family expansions and contractions varied across lepidopteran lineages and were associated with host plant use and specialization. Notably, they observed a higher expansion rate of detoxification-related gene families in Noctuidae, which they linked to the large number of species able to feed on various kinds of food in this clade.

While not all these studies explicitly used HOGs, the core principle—tracking orthologs and paralogs in a phylogenetic framework—remains the same. Databases with precomputed HOGs across thousands of genomes facilitate investigation of expansion and contraction patterns in any clade or gene family of interest.

### Ancestral Gene Order

Building on HOGs as proxies for ancestral gene content, HOGs can be used as a framework to infer ancestral gene order. By comparing gene order across extant species, one can reconstruct ancestral chromosomal arrangements and identify conserved or structurally rearranged regions. This approach is especially useful for studying karyotype evolution and genome architecture (Rascol et al. 2007).

AGORA (Algorithm for Gene Order Reconstruction in Ancestors) is a prominent method in this area (Muffato et al. 2023). The AGORA method incorporates conserved synteny and gene order into ancestral genome reconstruction by first inferring ancestral gene sets (i.e., groups of homologous genes) and gene trees for each family at each ancestral node in the species tree. After inferring ancestral gene sets, it uses conserved adjacencies across extant species to reconstruct ancestral gene orders, producing "CARs" (contiguous ancestral regions). Among the 624 ancestral vertebrate, plant, fungi, metazoan, and protist genomes reconstructed by AGORA, 183 were near-chromosomal level CARs. Contiguity declines for deeper nodes (> 100 Ma), but remains high for well-sampled clades like placental mammals. Interestingly, when assemblies with low contiguity are removed from the Boreoeutheria and Amniota datasets, the resulting ancestral reconstructions became worse: the reconstructed CARs were overall more fragmented and with lower quality adjacencies. This counter‐intuitive result is because even low-contiguity, fragmented assemblies still contribute true adjacency information and discarding them removes data that helps link conserved blocks in the ancestor.

When compared to the Ancestral Genomes database, AGORA produced highly similar gene content estimates for multiple key ancestors, which were comparable to the size of the genomes in the extant species (Muffato et al. 2023). Using 73 ancestral genomes and 74 extant vertebrates, the authors identified 5,749 intrachromosomal and 1,370 interchromosomal rearrangements across 5 billion years of evolution, with certain lineages such as Muridae and those including gibbon and dog showing exceptionally high rearrangement rates. Breakpoint hotspots were enriched near immune genes and depleted near developmental genes, suggesting functional constraints on genome structure (Muffato et al. 2023). AGORA makes these reconstructions publicly available through the Genomicus web database (Nguyen et al. 2022).

Another method that leverages HOGs for reconstructing ancestral gene order is EdgeHOG, a phylogenetically aware approach that propagates gene adjacencies along a species tree (Bernard et al. 2024). Once provided with HOGs and gene adjacencies from extant genomes, EdgeHOG parsimoniously propagates these adjacencies along the species tree, treating each HOG as an ancestral gene. This allows for the reconstruction of genomic contigs at multiple evolutionary depths while preserving syntenic relationships and accounting for duplications, losses, and rearrangements over time. Using this approach, EdgeHOG successfully reconstructed gene orders across diverse clades, including LECA. The results showed that many gene clusters thought to be unique to modern eukaryotes were already present in LECA, indicating deep evolutionary conservation of functional modules.

### Functional Annotation

Duplicated genes can undergo many fates, including neofunctionalization of one or both copies, conservation, subfunctionalization, pseudogenization, or specialization (Rastogi & Liberles 2005). Traditional orthology methods identify evolutionary relationships but lack resolution to track functions across nested duplications. HOGs offer a structured approach to mapping gene functions across multiple taxonomic levels, which allow for a more accurate prediction of gene function based on evolutionary history.

The fine-grained inference provided by HOGs allows researchers to study gene family functional divergence at different taxonomic levels. Take, for instance, the NADPH oxidase gene family in humans, which consists of at least seven members, each associated with distinct physiological roles due to subfunctionalization (Katsuyama et al. 2012). Examining this gene family within an unstructured orthology framework would merge all members into a single group, masking their distinct functions. In contrast, applying a HOG framework would capture the evolutionary relationships of these genes at multiple levels, which helps resolve their functional diversification.

Since orthologous genes tend to share the same function (Altenhoff et al. 2012; Gabaldón & Koonin 2013), HOGs offer a natural scaffold for propagating gene functions. Tools like HOGPROP, integrated into the QTLSearch algorithm (Warwick Vesztrocy et al. 2018), leverage the HOG hierarchy by propagating trait-associated annotations, e.g., GO or ChEBI terms. The method assigns confidence scores to genes within a Quantitative Trait Loci (QTL), based on their evolutionary relatedness to annotated genes, i.e., with consideration of speciation or duplication events. This allows one to rank candidate genes within each QTL region by their inferred functional relevance, thereby prioritizing those most likely to underlie the observed phenotype (Warwick Vesztrocy et al. 2018). This transfer of function through HOGs can also be used to find alternative bacterial model organisms tailored for specific biological processes of interest (Nicheperovich et al. 2022).

Other tools like PAINT (Phylogenetic Annotation and INference Tool) apply a similar logic, using curated gene trees to infer when functions were gained or lost (Feuermann et al. 2025; Gaudet et al. 2011). PAINT uses HOGs in the sense that it relies on curated gene family phylogenies to distinguish orthologs from paralogs. After phylogenetic tree construction, curators manually infer ancestral function gains and losses based on available experimental evidence from extant species. PAINT then propagates GO annotations across the tree to all genes that have inherited a function from their ancestor while blocking annotations for genes that have lost that function due to duplication or evolutionary divergence. The authors applied PAINT to resolve functional shifts following gene duplication and identify conserved functional modules across evolution. For example, PAINT revealed that the ancestral DNA mismatch repair gene MSH2 in LUCA was co-opted in vertebrates to regulate apoptosis and immunoglobulin hypermutation (Gaudet et al. 2011). More recently, PAINT was used in the PAN-GO study to manually curate the function evolution models, resulting in 68,667 integrated GO annotations of function for 17,079 human genes (Feuermann et al. 2025). This demonstrates the evolutionary flexibility of gene functions and how phylogenetic approaches improve functional annotation over flat orthologous groups.

### Phylogenetic Profiling for Finding Co-evolving Gene Families

Phylogenetic profiling predicts co-evolution by identifying shared patterns of presence, absence, duplication, or loss across species (Dey & Meyer 2015; Moi & Dessimoz 2023; Pellegrini et al. 1999). Genes that co-evolve—gained or lost together across the lineages—often participate in the same biological processes, protein complexes, or metabolic networks. This "guilt-by-association" approach has been used to infer novel gene functions, but traditional orthology methods lack the resolution needed to distinguish functional relationships at different evolutionary depths.

HOGs provide a more refined framework for phylogenetic profiling by explicitly incorporating duplication and loss events, which improves the resolution of co-evolutionary signals, particularly in large or complex families. Tools like HogProf (Moi et al. 2020) encode each HOG’s phylogenetic profile and allow for efficient comparison across thousands of species, allowing for high-throughput prediction of co-evolving genes. Phylogenetic profiles are increasingly integrated into HOG-based databases such as MBGD, eggNOG, and OMA (Altenhoff et al. 2024a, b; Hernández-Plaza et al. 2023; Uchiyama et al. 2025), making co-evolution analysis accessible at scale. These profiles support diverse applications, from inferring putative functional relationships between proteins to uncovering ancestral functional interactions.

## Methodology for Constructing Hierarchical Orthologous Groups

HOGs are inferred using diverse computational approaches that balance accuracy and scalability. Methods generally fall into three categories: graph-based clustering, gene tree-based inference, and hybrid approaches combining elements of both. To be considered as a true HOG framework, a method must follow a phylogenetic framework guided by the species tree, infer orthology hierarchically by capturing nested relationships at different taxonomic depths, and maintain hierarchical consistency across taxonomic levels. Here, we define hierarchical consistency as 1) all proteins belonging to a HOG at a lower level remain grouped within a single HOG at higher levels and 2) duplication events are consistently placed in evolutionary time across taxonomic levels.

Table 1 lists current computational approaches to HOG inference (to our knowledge, the list may not be exhaustive). Only actively maintained or recently published methods (within the last 10 years) are included. Methods are categorized according to their core inference strategy—graph-based, gene tree-based, or hybrid—and linked to the database implementing these methods. Note that gene tree-based methods typically do not explicitly label themselves as HOG approaches but implicitly produce HOGs through reconciled gene trees. Furthermore, we exclude methods like PhyloMCL (Zhou et al. 2020) and OrthoMCL (Li et al. 2003), which generate flat orthologous groups at a single taxonomic level. Constructing groups across multiple levels with these methods involves re-running the pipeline independently for each taxonomic clade, which does not guarantee hierarchical consistency.

**Table 1:** Overview of HOG methods

Method (algorithm)	Method type	Similarity search method	Clustering algorithm	Alignment method (for tree building)	Tree reconstruction method	Reconciliation method	Database name	References
OrthoDB (Ortho-Loger)	graph-based	MMSeqs2	Triangulating and clustering all RBHs and in-paralogs	N/A	N/A	N/A	OrthoDB https://www.orthodb.org	(Kriventseva et al. 2008, 2015; Kuznetsov et al. 2023; Tegenfeldt et al. 2024; Zdobnov et al. 2021)
OMA (GETHOGs 2)	graph-based	Smith-Waterman	GETHOGS (identifies connected components + MinCut)	N/A	N/A	N/A	Omabrowser https://omabrowser.org	(Altenhoff et al. 2013; Altenhoff et al. 2024a, b; Roth et al. 2008; C.-M. Train et al. 2017)
PhylomeDB V5	gene tree-based	BLAST	seed gene + BLAST to other genomes	MUSCLE, MAFFT, KALIGN, M-Coffee	Maximum likelihood IQ-TREE with different models. Best model selected with BIC	Species overlap	PhylomeDB https://phylomedb.org	(Fuentes et al. 2022; Huerta-Cepas et al. 2014)
PANTHER	gene tree-based	Mapping sequences to curated families using HMMs	Tribe-MCL	MAFFT	GIGA	GIGA	PANTHER https://pantherdb.org	(Mi et al. 2007, 2013; , 2017; Thomas 2010; Thomas et al. 2022)
Ensembl- Compara (Gene-Trees)	gene tree-based	BLAST + Smith-Waterman	hcluster_sg for phylogeny-aware clustering	M-Coffee or MAFFT for large groups	TreeBeST	TreeBeST	EnsemblCompara https://www.ensembl.org/info/genome/compara	(Herrero et al. 2016; Vilella et al. 2009)
Hieranoid 2	hybrid	BLAST, U-SEARCH possible	InParanoid	MUSCLE	Neighbor-Joining tree with Belvu	ETE3	HieranoidDB https://hieranoidb.sbc.su.se	(Kaduk & Sonnhammer 2017; Remm et al. 2001; Sonnhammer & Östlund 2015)
OMA (FastOMA)	hybrid	Mapping sequences to HOGs with OMAmer; linclust for unmapped seqs	GETHOGs 2	MAFFT	FastTree	Species overlap	N/A	(Majidian et al. 2025)
eggNOG 6.0	hybrid	Mapping sequences COGs with eggNOG mapper; SIMAP on Smith-Waterman RBHs for unmapped seqs	(COG) triangulating and clustering all RBHs	MUSCLE, M-Coffee, Clustal Omega, MAFFT	FastTree, PhyML	ETE3	eggNOG http://eggnog6.embl.de	(Hernández-Plaza et al. 2023; Huerta-Cepas et al. 2016a, b; Powell et al. 2012, 2014)
OrthoFinder 2.0	hybrid	BLAST	MCL	MAFFT	DendroBLAST + STAG + STRIDE	Species overlap + duplication-loss- coalescent model	N/A	(Emms & Kelly 2015, 2019)
MBGD	hybrid	DIAMOND for pangenome construction; BLASTP + SW for top-level ortholog grouping	DomClust + DomRefine (UPGMA domain-aware clustering)	FAMSA	FastTree	DomRefine	MBGD https://mbgd.nibb.ac.jp	(Uchiyama 2006; Uchiyama et al. 2025)

### Graph-Based HOG Methods

Graph-based methods construct HOGs using sequence similarity graphs, where genes are nodes connected by edges reflecting sequence similarity. These approaches rely on graph theory to model similarity relationships between genes, often using variations of the reciprocal best hit (RBH) concept—also referred to as bi-directional best hits or reciprocal smallest distances. Such “nearest neighbor” methods approximate orthology by assuming that the most similar pairs of genes across genomes are likely orthologs (Kuzniar et al. 2008). Typically, these methods begin by comparing protein sequences, which allow capturing broader evolutionary distances compared to nucleotide sequences. An all-against-all similarity search is conducted using algorithms such as BLAST (Altschul et al. 1990), Smith-Waterman (Smith & Waterman 1981), MMSeqs2 (Steinegger & Söding 2017), or DIAMOND (Buchfink et al. 2021), with alignment parameters such as percentage overlap influencing the sensitivity and specificity of matches. Orthologous relationships are often followed by the inclusion of recent in-paralogs—genes within the same genome that are more similar to each other than to any gene in another genome.

Subsequent clustering algorithms group genes into orthologous clusters based on this information. Approaches vary among methods: OrthoDB triangulates and clusters RBHs and in-paralogs hierarchically; OMA identifies orthologous groups by computing connected components in similarity graphs, then applies iterative minimum-cut (MinCut) partitioning to refine groups; other methods may employ Markov clustering (MCL) for grouping genes based on graph structure. Hierarchical relationships are then introduced by applying taxonomic constraints from a known species tree, making sure that orthologous groups are organized consistently across multiple taxonomic levels.

**Fig. 6:**
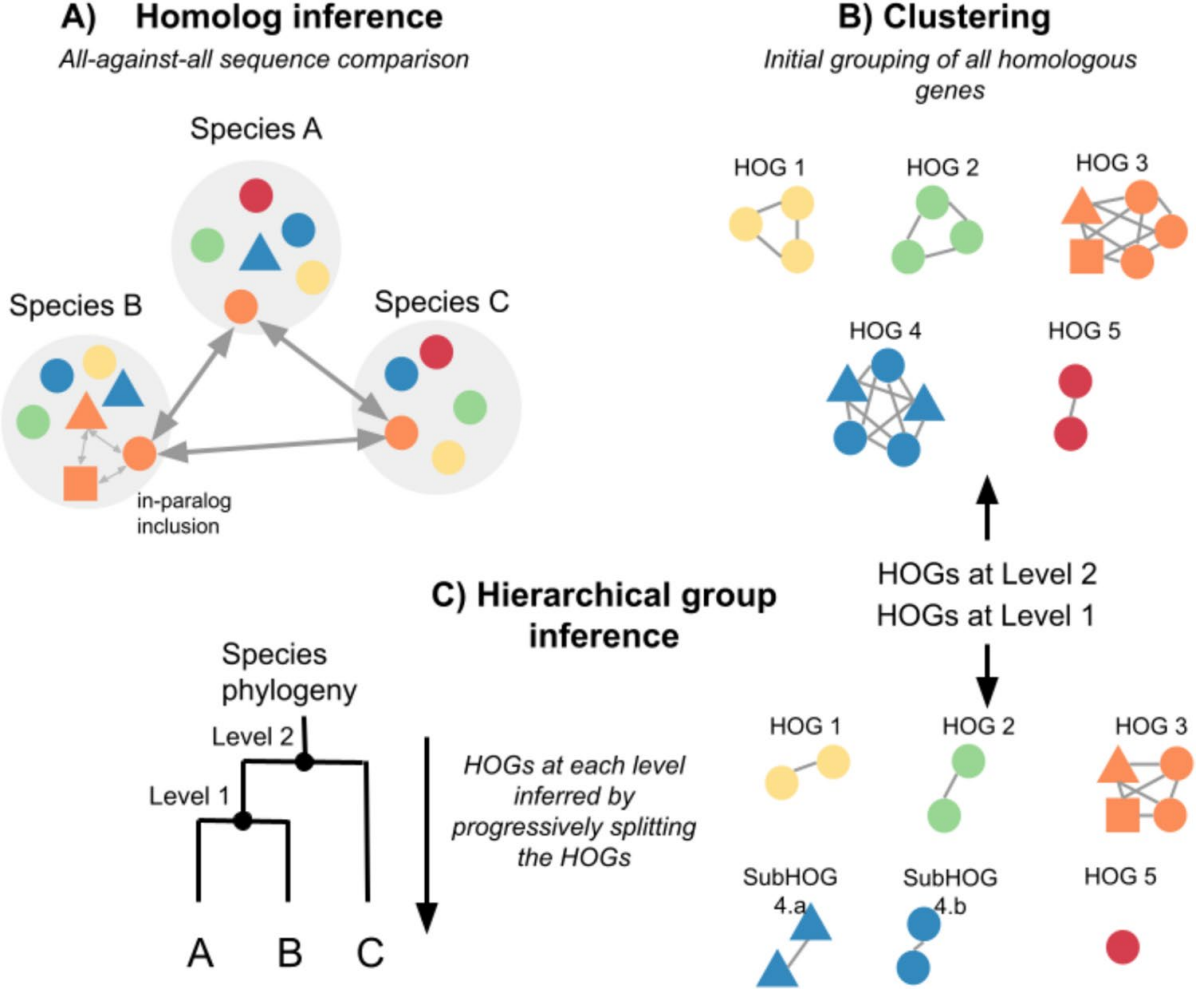
Top-down graph-based HOG inference. **A** To infer homology all sequences are compared against each other. Generally, orthologs are determined by reciprocal best hits between genomes (large arrows), and in-paralogs are determined as genes in the same genomes having a smaller evolutionary distance or score than orthologs (small arrows). **B** Homologous genes are clustered together using an iterative min-cut algorithm to infer HOGs. **C** HOGs are progressively subdivided to smaller ones guided by species tree, inferring subHOGs at each level

The OMA GETHOGs algorithm (Altenhoff et al. 2013) initially employed a "top-down" clustering approach by subdividing groups starting at the root of the species tree and progressing to the leaves (Fig. 6). This was later improved in GETHOGs 2 (C.-M. Train et al. 2017), which instead uses a “bottom-up” strategy by merging clusters progressively from the leaves upward. This improves robustness and avoids error propagation from higher taxonomic levels. OrthoDB (Kuznetsov et al. 2023) similarly uses a bottom-up hierarchical clustering approach, merging orthologous clusters hierarchically along the species tree.

A significant advantage of graph-based approaches is their scalability, as sparse similarity networks allow efficient handling of large genome datasets without requiring full phylogenetic reconstructions. However, these methods are sensitive to parameter choices, such as sequence similarity thresholds, clustering inflation parameters, and edge weighting strategies.

### Gene tree-based HOG Methods

Gene tree-based approaches explicitly reconstruct phylogenetic trees for gene families and reconcile them with a known species tree (Altenhoff et al. 2024a, b; Nevers et al. 2022). These methods distinguish speciation events from duplication events within gene trees, thus inherently producing evolutionarily consistent hierarchical orthologous groups. Correctly inferred gene trees provide high accuracy in delineating orthology and paralogy, overcoming some limitations of graph-based methods. A classical example is the LOFT algorithm (van der Heijden et al. 2007), which systematically assigns orthology levels based on speciation and duplication nodes in gene trees.

Gene tree-based pipelines generally proceed as follows: First, initial homologous clusters (coarse-grained orthologous groups) are identified, typically using similarity search algorithms like BLAST and clustering algorithms such as hcluster_sg, employed by EnsemblCompara (Herrero et al. 2016). Following clustering, gene tree-based and hybrid approaches usually perform multiple sequence alignment using standard software such as MAFFT (Katoh & Standley 2013), MUSCLE (Edgar 2004), KALIGN (Lassmann 2019), or M-Coffee (Wallace et al. 2006); PhylomeDB notably combines all these alignment methods for robustness (Fuentes et al. 2022). Multiple sequence alignments are often filtered to remove columns with excessive gaps. For example, PANTHER removes columns aligning fewer than 75% of sequences, employing a weighted percentage to reduce phylogenetic bias (Mi et al. 2013). Next, phylogenetic tree construction and reconciliation are performed. PhylomeDB, for instance, uses IQ-TREE under maximum likelihood frameworks and compares various evolutionary models to select the best-fitting one based on Bayesian Information Criterion (Fuentes et al. 2022). Tools often use FastTree (Price et al. 2010) for rapid tree reconstruction, as in FastOMA and MBGD (Majidian et al. 2025; Uchiyama et al. 2025). Notably, OrthoFinder first infers unrooted gene trees using DendroBLAST, infers rooted gene trees using STAG and STRIDE (Emms & Kelly 2017, 2018), then reconciles the gene trees using a duplication-loss-coalescence model (Emms & Kelly 2019). Other pipelines integrate specialized tree-building and reconciliation algorithms directly: PANTHER employs the GIGA algorithm (Thomas 2010), and EnsemblCompara uses TreeBeST (Vilella et al. 2009), both specifically designed to simultaneously infer gene trees and reconcile them to the species tree. Other methods such as species overlap are commonly used for reconciliation.

However, gene tree-based methods are computationally demanding and require both reliable rooting and high-quality alignments. Midpoint rooting, though widely used, can be misleading in families with heterogeneous evolutionary rates, and manual outgroup selection is difficult to scale across thousands of gene trees. An alternative strategy is to root gene trees by using heuristics to choose a gene tree that minimizes the dissimilarity to the species tree (Kuzniar et al. 2008; Page & Charleston 1997). In addition, tree topology depends critically on the quality of the multiple sequence alignment used to build it. Misaligned regions can produce erroneous branching patterns, leading to incorrect orthology or paralogy assignments.

### Hybrid HOG Methods

Hybrid approaches combine elements of both graph-based and tree-based methods to balance computational efficiency with evolutionary accuracy. In this review, we define hybrid methods strictly as those that combine graph-based clustering with explicit phylogenetic gene tree construction and reconciliation; the use of a species tree alone does not qualify, as it is inherent to all HOG inference. Hybrid methods often begin with initial clustering steps, similar to previously described graph-based methods, followed by phylogenetic reconciliation akin to those employed by gene tree-based methods to refine hierarchical relationships (see Table 1).

OrthoFinder (Emms & Kelly 2015, 2019), for example, initially relied on graph-based clustering with MCL but later incorporated gene tree construction. Other initial clustering techniques in hybrid approaches use HOGs defined with other orthology software, e.g., Hieranoid, which uses InParanoid for clustering (Kaduk & Sonnhammer 2017) and FastOMA, which relies on precomputed HOGs in the OMA database made using GETHOGs (Majidian et al. 2025). MBGD, tailored for microbial genomes, uniquely integrates domain-aware clustering using the DomClust algorithm (Uchiyama 2006; Uchiyama et al. 2025). Some hybrid methods, including eggNOG and FastOMA, employ mapping strategies for initial clustering and are described in more detail in the next section.

Many methods we described as “tree-based” or “graph-based” in fact integrate elements of both, resulting in hybrid approaches. For example, tree-based methods typically rely on graph-based clustering to define input gene families, while graph-based methods often incorporate phylogenetic information via hierarchical expansion across the species tree. The choice of methodology for HOG inference depends on the specific research objectives and the scale of the dataset. Graph-based methods are highly efficient and scalable, making them ideal for large comparative genomic studies, but they can be sensitive to parameter choices and may struggle to accurately resolve complex evolutionary histories. Tree-based methods offer greater biological accuracy by explicitly modeling gene family evolution, but they are computationally demanding and less scalable. Hybrid approaches aim to strike a balance between scalability and evolutionary accuracy by leveraging the strengths of both methodologies; therefore, most recent HOG methods are trending toward hybrid strategies.

### Mapping Query Genes to HOGs

An important complementary strategy used in certain HOG methods is mapping query sequences directly to existing HOGs present in a given orthology database. Rather than re-running orthology inference from scratch, which is computationally inefficient, these methods take a more practical solution and place novel query sequences into pre-defined HOGs without changing the HOGs.

This mapping involves two main steps. First, the query sequence must be assigned to the correct known protein family (represented by the deepest-level HOG). This initial placement is often performed by indexing all families in advance and running search algorithms like DIAMOND, MMSeqs2, or HMMER (Eddy 2009). Once a family is identified, the precise placement of the query within the HOG is determined. This step can be achieved with methods for phylogenetic placement, such as pplacer (Matsen et al. 2010), EPA-ng (Barbera et al. 2019), APPLES (Balaban et al. 2022), and EPIK (Romashchenko et al. 2023). Originally designed for taxonomic and diversity analyses of metabarcoding data (Czech et al. 2022), phylogenetic placement methods were adopted for orthology by tools like TreeFam (Schreiber et al. 2014), TreeGrafter (Tang et al. 2019), and SHOOT (Emms & Kelly 2022).

Tools such as TreeGrafter, employed by the PANTHER database, leverage these phylogenetic placement methods to integrate novel queries into established subfamilies. Other methods like eggNOG-mapper (Cantalapiedra et al. 2021) and ODB-mapper (Tegenfeldt et al. 2024) use mapping approaches and assign the query to the best matching orthologous group directly based on the results of sequence searches against hierarchical clusters. Similarly, FastOMA employs OMAmer, which implements an alignment-free algorithm based on k-mer matches to rapidly map query sequences to existing HOGs (Rossier et al. 2021).

## Open Challenges in HOG Inference

HOGs have emerged as a powerful framework for studying gene family evolution across multiple taxonomic scales. However, despite their growing adoption, several challenges remain in their inference and application. Creating robust methods to infer accurate HOGs which reflect the gene families’ real evolutionary history is equally as important as creating scalable methods to deal with the influx of genomic sequence data.

### Dependence on Underlying Orthology Inference

The accuracy of HOG inference fundamentally depends on the quality of the underlying orthology inference. Differences between orthology methods or parameter settings can yield substantially different results, leading to variability in HOG composition. Domazet-Lošo et al. (2024) demonstrated that varying the clustering parameter *c* in MMseqs2 drastically changes the number and composition of inferred gene families. This parameter controls the minimum fraction of alignment overlap required for sequences to be grouped into the same cluster (Steinegger & Söding 2017). A higher *c* value enforces stricter clustering, ensuring that sequences within a cluster share a more conserved overall domain architecture, while a lower *c* value allows more divergent sequences to be grouped together. In this study, a stringent clustering threshold (high *c*) leads to more, smaller HOGs, whereas a permissive threshold (low *c*) results in fewer, larger HOGs that encompass more sequence diversity. For certain clades, the number of inferred gene families fluctuates dramatically depending on *c*, highlighting how parameter choices in orthology inference can result in different estimations of ancestral genome content.

This point is further evidenced by the choice of similarity metric used to weight edges in similarity graphs, such as BLAST percent identity, e-value, raw score, or bit score, which can also influence clustering outcomes. In a comparative analysis, Kuzniar et al. (2008) found that while 77-88% of orthologs were consistently inferred across similarity metrics, a substantial fraction varied depending on the metric used.

Moreover, detecting deeply homologous genes that have diverged significantly over long evolutionary timescales poses an additional challenge. Highly divergent genes may evade detection by conventional sequence-based approaches (Rost 1999). Overcoming this issue may require integrating additional evidence, such as structural, syntenic, or functional information, to refine the detection of ancient homologs (Boubaker et al. 2023).

### Dependence on Annotation and Assembly

Errors or gaps in genome annotations and assemblies significantly impact HOG accuracy by propagating into the orthology inference. A study by Prieto-Baños et al. (2025) found that discrepancies among annotation method results using the same assemblies impact orthology inference (comparing Ensembl, NCBI, UniProt, and the *ab initio* method Augustus). Differences in protein length, potentially due to gene fragmentation, and misannotations contributed to variation in HOG number, composition, and accuracy. Incomplete or fragmented gene annotations can artificially split gene families or suggest incorrect gene losses. Indeed, a benchmark by Trachana et al. (2011) showed that outdated or inaccurate gene models contributed to nearly 40% of observed errors in orthology predictions, which underscores the critical influence of genome annotation quality on orthology inference.

Furthermore, Prieto-Baños et al. found that using the longest isoforms compared to the evolutionarily best conserved isoforms resulted in differences in orthology benchmarking results (2025). The results suggest that isoform choice significantly influences the multiple sequence alignments, gene tree topologies, and thus the delineation of orthologs and paralogs. Standardizing gene annotations to the most biologically meaningful canonical isoforms would help to alleviate this problem, and several tools have implemented strategies to define a single main protein isoform based on evolutionary or length conservation, such as APPRIS (Rodriguez et al. 2022) MANE Select (Morales et al. 2022), OMA (Altenhoff et al. 2021), ortho2tree (Insana et al. 2024), PALO (Villanueva-Cañas et al. 2013), or IsoSel (Philippon et al. 2017). For example, IsoSel automatically selects the single protein isoform per gene that maximizes the alignment quality across species. The authors reconstructed gene trees for 154 eukaryotic families using IsoSel-selected isoforms versus longest or random isoforms and found that the resulting topologies differed substantially: trees made from IsoSel isoforms were consistently more congruent with a trusted species tree (as evidenced by lower duplication-loss reconciliation scores) (Philippon et al. 2017). This indicates that the choice of isoforms can meaningfully change the inferred topology of gene trees and thus impact orthology inference. Ultimately, applying such canonical isoform definitions across genome sequencing initiatives would result in standardized annotations and in turn better orthology inference.

Contamination, such as bacterial or fungal sequences that are inadvertently included in eukaryotic genomes, can significantly distort HOG inference for the gene families including contaminant species. This contamination artificially inflates the inferred evolutionary depth of affected HOGs, making them appear as if they originated much earlier than they actually did. This could lead to erroneous conclusions about gene emergence and ancestral genome content. Addressing this issue requires careful contamination filtering with tools like BlobToolKit (Challis et al. 2020) before HOG construction, or the use of orthology inference methods that distinguish between genuine orthologs and spurious matches from foreign DNA.

Tools such as OMArk (Nevers et al. 2024) and BUSCO (Manni et al. 2021) use precomputed orthologous groups to assess the quality of proteomes, with OMArk leveraging HOGs to estimate the expected ancestral protein content at a given taxonomic level, and BUSCO using single-copy orthologous genes. These tools can identify problematic annotations prior to orthology inference. OMArk can also be used to evaluate contamination levels (Nevers et al. 2024).

### Dependence on Species Phylogeny

HOG inference assumes a known and accurately rooted species tree, thus, errors in the phylogeny can propagate through the HOG framework. Indeed, efforts to combine phylogenetic studies have uncovered regions of the tree that show substantial conflict (e.g., (Hinchliff et al. 2015)). Misplaced or unresolved branches can lead to artificial clustering or fragmentation of HOGs and result in erroneous inferences of duplications or losses. Additionally, events such as incomplete lineage sorting, hybridization, or horizontal gene transfer (HGT) can shape genome evolution in ways that are not fully captured by a bifurcating species tree. Even when the species tree is correct and fully resolved, it may still fail to capture the full evolutionary history of individual genes. Speciation is not instantaneous but occurs over extended periods in populations, during which duplications can arise in ancestral lineages that are not represented as discrete branches in the tree. This creates a scenario similar to polytomies: some duplications may appear to map to extant lineages but actually took place in unsampled or collapsed ancestral populations. Theoretically, small perturbations in the species tree topology can lead to vastly different numbers and composition of HOGs.

Some methods account for species tree uncertainty by integrating alternative strategies for species tree construction and rooting. For example, OrthoFinder infers and then roots the species tree using its STAG (Species Tree from All Genes) and STRIDE (Species Tree Root Inference from gene Duplication Events) algorithms, which are designed to handle gene duplication and loss while leveraging phylogenetic signal across multiple gene trees​ (Emms & Kelly 2017, 2018, 2019). Another approach is to permit multifurcating species trees, which accommodate polytomies where phylogenetic relationships are unresolved, and is implemented in tools such as FastOMA (Majidian et al. 2025). However, this strategy affects HOG inference by potentially inflating the number of groups or reducing resolution at deeper taxonomic levels​. Future improvements might incorporate confidence measures on species tree topologies and demand a certain minimum level of support for nodes to be used for inferring HOGs for the databases or use ensemble approaches that integrate multiple plausible phylogenies to better capture uncertainty in evolutionary histories.

### Dependence on Evolutionary Assumptions

HOG inference relies on fundamental evolutionary assumptions that, while useful, introduce biases that shape the resulting orthologous groups. Most approaches treat genes as the primary evolutionary unit, yet protein evolution often occurs at the domain level through fusions, fissions, and domain shuffling. Such domain-level events can create discrepancies between gene-based orthology and true evolutionary relationships (Bornberg-Bauer et al. 2010; Forslund & Sonnhammer 2012). This limitation can lead to artificial fragmentation of gene families or the incorrect merging of functionally distinct genes. The need for domain-level orthology inference has already been recognized (Dessimoz et al. 2012; Forslund et al. 2018; Linard et al. 2021; Sonnhammer et al. 2014), yet relatively few tools have implemented it in the context of hierarchical orthology frameworks. Notably, Domainoid (Persson et al. 2019), SonicParanoid 2 (Cosentino et al. 2024), and MBGD (Uchiyama 2006) have explicitly incorporated domain-level orthology into their methods (albeit Domanoid and SonicParanoid 2 are not HOG methods).

Additionally, HOG inference presumes vertical descent. However, HGT, which is common in prokaryotes and documented in some eukaryotic lineages, violates this assumption. Most HOG inference methods do not explicitly model HGT, potentially leading to misclassified orthologous relationships due to sequences resulting from HGT appearing close to each other on the gene tree. This results in an added complication to orthology inference, as it needs specialized phylogenetic or parametric methods to detect HGT (Ravenhall et al. 2015). PANTHER is one exception that integrates gene transfer events into its evolutionary model (Thomas et al. 2022).

### Differences Among Databases

Differences in HOG inference pipelines, ranging from species sampling, orthology algorithms, parameter choices, and evolutionary assumptions can result in major discrepancies between databases.

**Fig. 7:**
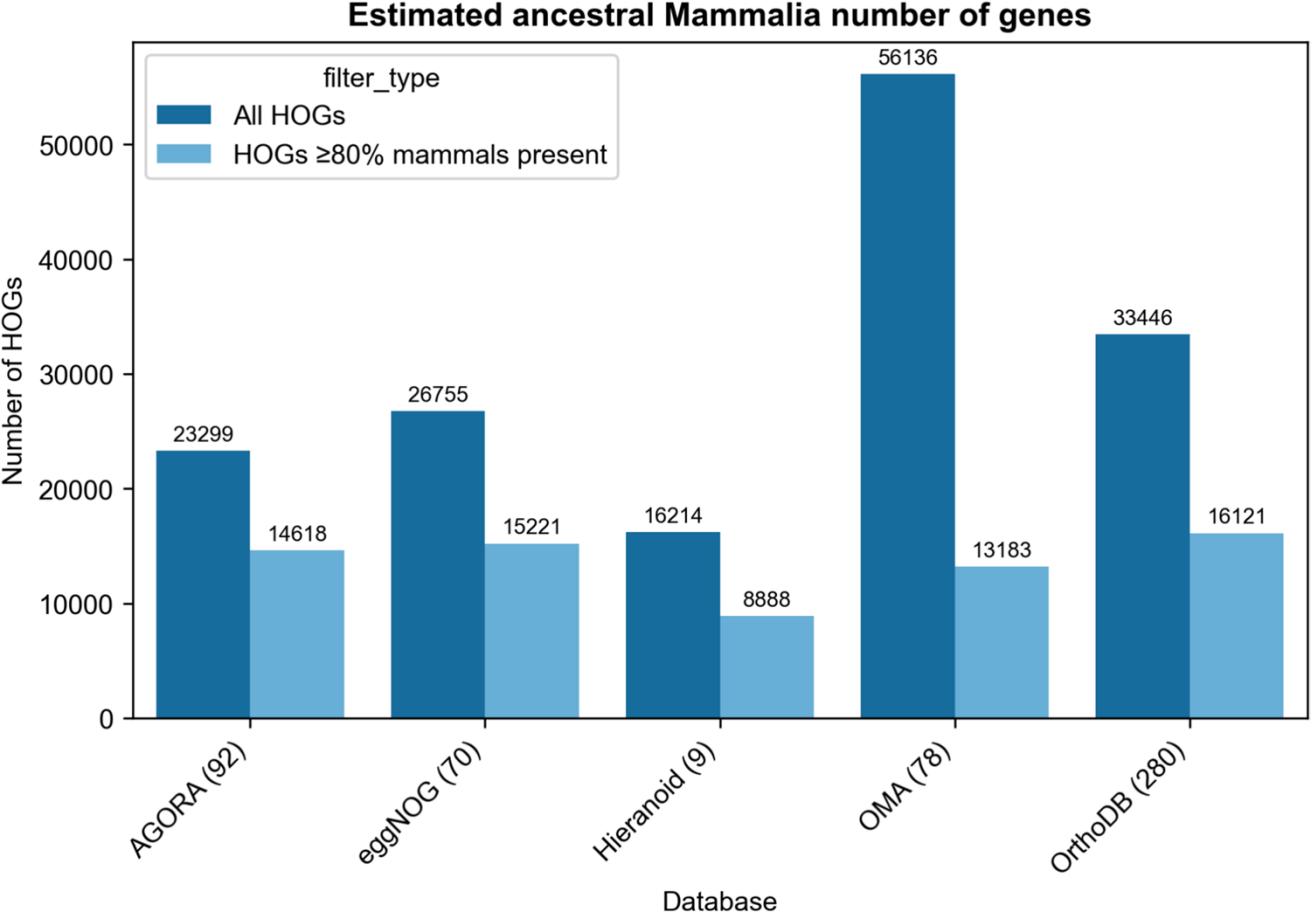
Comparison of estimated conserved ancestral gene repertoire size, i.e., number of HOGs, at the Mammalian level. Data were collected from AGORA < https://www.genomicus.bio.ens.psl.eu/ > , eggNOG < http://eggnog6.embl.de/ > , Hieranoid < https://hieranoidb.sbc.su.se/ > , OMA Jul2024 release < https://omabrowser.org/ > , and OrthoDB v12.1 < https://www.orthodb.org/ > . For each database, two values are given: the total number of inferred HOGs (unfiltered) and HOGs filtered at 0.8 completeness, meaning that at least 80% of the mammalian species present in the database are represented in the HOG (Altenhoff et al. 2024a, b). The number of mammalian species for each database is shown in parentheses. All data were collected from the online versions of the databases in July of 2025 (see Supplementary notebook)

As an illustration, we compared several HOG databases (Genomicus, which uses the AGORA algorithm, eggNOG, Hieranoid, OMA, and OrthoDB) to estimate the conserved ancestral gene repertoire size at the Mammalia level, defined as the number of HOGs assigned to the mammalian common ancestor (Fig. 7). The inferred counts ranged from 16,214 in Hieranoid to 56,136 in OMA. The notably high count in OMA may reflect an excess of small, potentially spurious HOGs containing only a few genes, possibly arising from contamination in structural annotations or over-splitting due to shared domains.

To reduce such noise, we applied a completeness-based filter and retained only those HOGs containing ≥ 80% of the mammalian species represented in each database (i.e., "Completeness score" in (Altenhoff et al. 2024a, b)). After filtering, the estimates converged: four out of five databases yielded HOG counts between 13,183 and 16,459, suggesting this range may be a more robust estimate of the conserved ancestral gene repertoire size. Hieranoid was the outlier, with only 8,888 filtered HOGs, likely due to its limited taxon sampling (only 9 mammalian species) compared to over 70 in the other databases. With so few species, the phylogenetic diversity may be insufficient to capture the full repertoire of ancestral gene families. Moreover, the filtering may have been too stringent in Hieranoid: with only 9 mammalian species, even one missing member disqualifies a HOG from passing the 80% threshold. That said, adding more genomes beyond a critical number and phylogenetic spread may yield diminishing returns for estimating ancestral gene repertoire. This comparison highlights the importance of both filtering and taxon selection in estimating ancestral genome content.

Overall, differences in inferred HOG numbers across databases reflect not only filtering thresholds and taxon sampling but also likely other methodological distinctions such as parameter choice for homology/orthology detection, the species phylogeny used as input, or the underlying gene annotation quality. It also underscores the need for accessible and transparent HOG resources: in this small analysis, we only included databases where the necessary information could be readily retrieved through a web interface or parsed with simple scripts (see supplementary Jupyter notebook). In many other resources, obtaining such counts is not straightforward. Understanding these factors is essential when comparing HOG-based analyses across studies.

### Scalability of HOG Inference

Methods for HOG inference inherit the scalability challenges common for orthology inference in general. Running time and memory requirements are heavily influenced by the number of genomes analyzed, the total number of sequences, and the size of corresponding gene families.

One significant core problem is to establish homology between different sequences of input genomes. This often implies an all-against-all de novo comparison for graph-based and hybrid methods such as OrthoDB, OMA GETHOGs, OrthoFinder (Altenhoff et al. 2013; Emms & Kelly 2019; Kriventseva et al. 2019) and others. Tree-based methods also require this step during the clustering that precedes multiple sequence alignment (Fuentes et al. 2022; Vilella et al. 2009). Because of this, running time of traditional orthology inference is typically a power-law relationship with the number of input species (Emms & Kelly 2019; Majidian et al. 2025). With the growing number of sequence data available, it quickly becomes intractable if the numbers of species are in the thousands.

Different heuristics were developed to circumvent the need for exhaustive sequence comparisons and to achieve linear complexity in the number of input genomes. For example, Hieranoid (Kaduk & Sonnhammer 2017) traverses a linear number of species tree nodes and compares only neighboring clades, which are represented by consensus sequences. SonicParanoid (Cosentino et al. 2024) speeds up alignments with machine learning, although its running time remains quadratic. FastOMA (Majidian et al. 2025) first maps input sequences to pre-defined orthologous groups with a scalable alignment-free method, and only unmapped sequences are clustered *de novo*.

Another major challenge lies in the construction of gene trees. Accurate tree reconstruction can be achieved with maximum likelihood methods under models of sequence evolution; however, these methods scale super-exponentially with the number of taxa (Stamatakis & Kozlov 2020). To address this, orthology methods rely on heuristics that limit tree optimization considerably to trade-off accuracy for speed. For example, some reconstruct the tree topology with distance-based inference but optimize the branch lengths with maximum likelihood (Huerta-Cepas et al. 2011). Others opt for approximate maximum likelihood methods (see Table 1) such as FastTree (Price et al. 2010). FastOMA additionally accelerates tree construction by subsampling sequences from gene families (Majidian et al. 2025).

### Lack of tools to Extract Information from HOGs

**Fig. 8:**
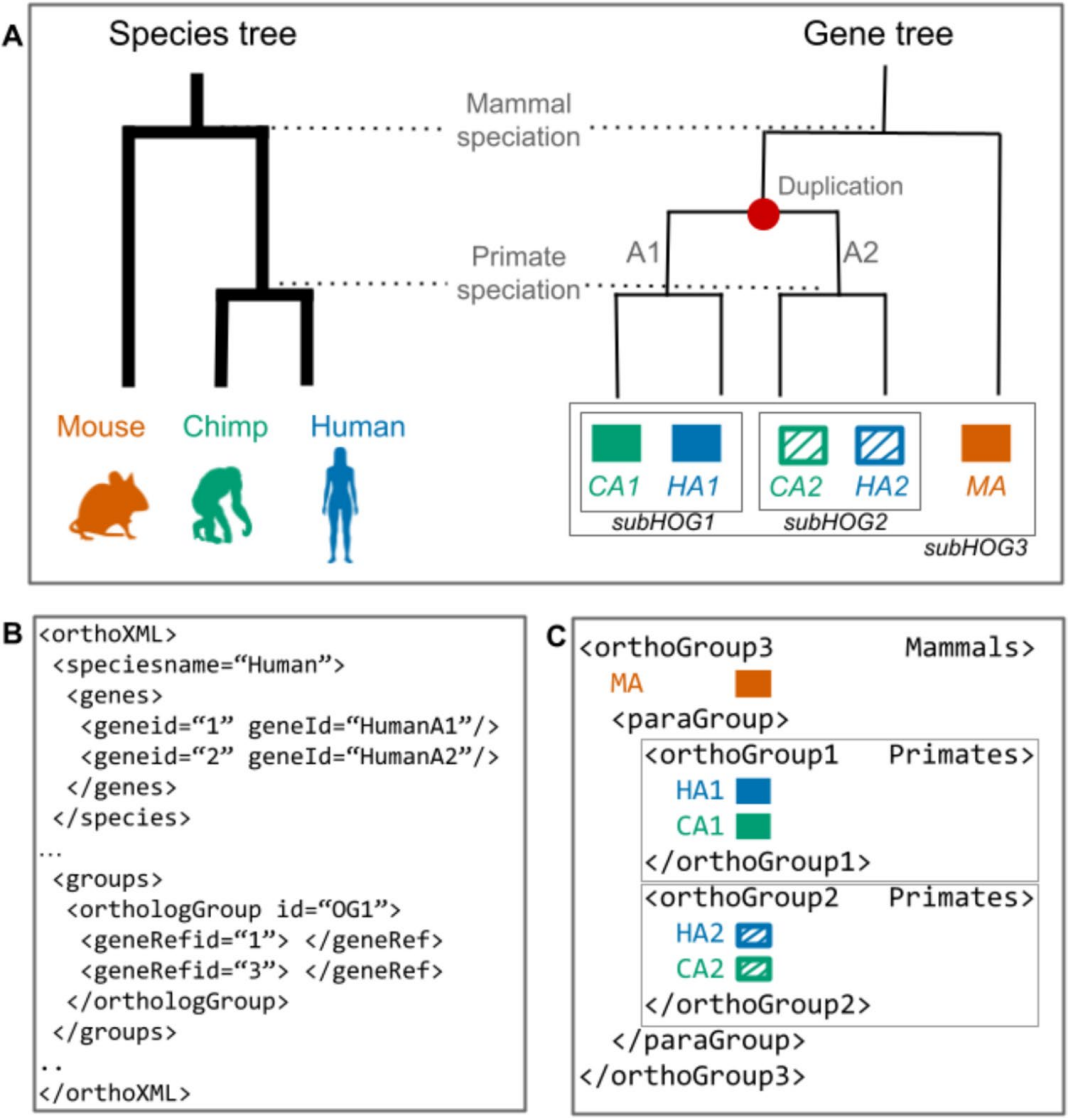
Example of an Ortho-XML file format and how it encodes the nested HOGs (Figure adapted from (Majidian et al. 2025)). **A** Schematic representation of a species tree and gene tree, relating a mouse gene co-orthologous to two chimp and two human genes, resulting from a duplication event preceding the Primate speciation. HOGs are delineated by boxes. **B** An excerpt of an OrthoXML file where gene IDs are listed for each species, followed by delineation of groups. Groups may be ortholog groups or paralog groups. Here, Ortholog Group 1 is shown, which contains the human A1 gene and the Chimp A1 gene, represented with their OrthoXML integer IDs defined in the OrthoXML header. **C** The nested pattern of the HOGs is reflected in the OrthoXML, as shown in this example for OrthoGroup 3. The primate-specific duplication is shown as a < paraGroup > , which then contains the two < orthoGroups > resulting from this duplication

OrthoXML was introduced as a standardized format to facilitate the exchange and integration of orthology data across different databases and tools (Fig. 8) (Schmitt et al. 2011). It has been widely adopted by orthology inference methods, including OMA, Ensembl, and HieranoidDB, making it a central format for encoding HOGs. Despite its structured design, OrthoXML is not easily human-readable, and working with it often requires custom scripts or dedicated parsing libraries.

Several tools exist to facilitate this, such as pyHam, a Python-based library that allows users to extract HOG composition, track duplications and losses, and visualize gene family evolution from OrthoXML files​ (C.-M. Train et al. 2019). Other options include the OrthoXML parser etree2orthoxml in the ETEToolkit (Huerta-Cepas et al. 2016a, b), Matreex for copy number analysis (Rossier et al. 2024), or the new OrthoXML-tools, described in this issue (Yazdizadeh Kharrazi et al. 2025). Still, all of these require programming knowledge, which limits their accessibility for researchers without a computational background.

### The Need for HOG-Based Benchmarks

The Quest for Orthologs (QfO) initiative has led benchmarking efforts for orthology inference by establishing standardized tests to assess their accuracy (Altenhoff et al. 2016, 2020; Altenhoff et al. 2024a, b; Nevers et al. 2022). These benchmarks evaluate inference methods on a set of 81 UniProt Reference Proteomes by measuring their precision and recall against a variety of tests, including species tree discordance tests, gold-standard gene tree tests, and function-based tests. However, these evaluations are tailored to pairwise orthology relationships and do not capture the hierarchical structure of HOGs.

Currently, the QfO benchmarking platform does not directly use the HOG structure in OrthoXML format, as they are flattened into gene pairs for evaluation. This flattening ignores duplication-aware structure. Thus, it treats all gene pairs within a group as orthologous (unless from the same species), potentially including in-paralogs after the LCA of all the genomes as false positives. This undermines the strength of the HOGs: their ability to distinguish orthologs from out-paralogs across taxonomic levels. Additionally, there are no QfO benchmarks to evaluate paralogy inference, duplication timing, or the hierarchical nesting of the groups. Even when HOGs are reduced to pairs of orthologs, some existing benchmarks are not optimized for HOG evaluation, such as the Generalized Species Tree Discordance test (GSTD) provided by the QfO Orthology Benchmarking Service (Altenhoff et al. 2016). This test first samples gene orthologs from sister clades and measures recall as the proportion of gene families that include orthologs from all species under consideration. It then reconstructs gene trees from these sampled orthologs and compares them to the reference species tree using the Robinson–Foulds (RF) distance to estimate precision.

However, gene trees do not always reflect the actual evolutionary relationships of organisms or the true gene evolutionary histories. From a methodological standpoint, gene trees are estimated from finite and often noisy sequence data and are vulnerable to stochastic or systematic errors such as taxon sampling, locus sampling, model misspecification, long-branch attraction, MSA errors, and biases such as heterogeneous evolutionary rates, saturation, heterotachy, or compositional bias (Braun et al. 2019; Kapli et al. 2021; Steenwyk et al. 2023). For example, shifts in GC content and lineage-specific rate variation can distort tree topologies even for conserved, single-copy genes. A striking case is the brain-derived neurotrophic factor (BDNF) gene in birds: although BDNF is well-conserved and not known to have undergone duplication or loss, its inferred gene tree is highly discordant with the avian species tree. This discordance is attributed to changes in base composition and evolutionary rate that mislead standard phylogenetic inference methods (Braun et al. 2019). In addition to methodological artifacts, real biological processes can also cause incongruence between gene trees and species trees such as incomplete lineage sorting, HGT, hybridization or introgression, recombination, asymmetrical gene duplication and loss, or convergent evolution (Steenwyk et al. 2023).

**Fig. 9:**
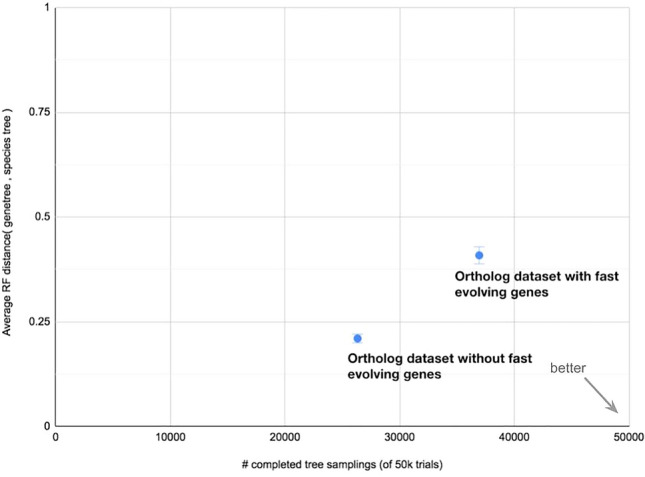
Effect of fast-evolving genes in simulated genomes, as benchmarked with the Generalized Species Tree Discordance test. The addition of fast-evolving genes caused a decrease in accuracy (average RF distance, y-axis), but an increase in recall (number of complete gene trees out of 50,000 trials, x-axis) (C. M. Train 2019)

Including more sequences by expanding the taxon set can potentially help to increase recall by capturing more orthologs, but it also increases the likelihood of including genes with unusual lineage-specific characteristics that complicate gene tree inference, such as the aforementioned shifts in base composition or evolutionary rate. Benchmarking based on tree concordance (e.g., RF distance) is therefore limited because it cannot distinguish between error introduced by orthology inference and genuine biological complexity. For example, simulations of HOGs containing genes with different rates of evolution illustrate that including fast-evolving, divergent sequences in HOGs increased recall in the GSTD benchmark (by recovering more complete orthologous sets), yet, it simultaneously decreased precision due to higher RF distances ((C. M. Train 2019); Fig. 9). This drop in precision is not necessarily a failure of the HOG reconstruction, but rather a limitation in the benchmark; the RF score is sensitive to errors in gene tree reconstruction, which become more pronounced with highly divergent sequences. As a result, the GSTD test may penalize more complete, biologically valid HOGs.

Thus, there remains an important distinction between what can be achieved through large-scale computational inference and what can be learned from detailed gene-by-gene examination. Even with high-quality proteomes and efficient HOG inference tools that perform well overall, certain gene families, particularly those with complex evolutionary histories, may require manual inspection to fully resolve convoluted events and achieve the highest quality orthology inference. This balance between tree-of-life-scale HOG inference using computational methods and resolution reflects the inherent complexity of the evolutionary processes that HOGs are designed to capture and highlights the need for benchmarks that account for inherent difficulties in gene tree inference, especially under realistic evolutionary scenarios.

Furthermore, a key factor in such benchmarking comparisons is the chosen reference species tree. While many parts of the tree of life are well resolved and of high quality, every reference tree remains an estimate (as discussed in the Dependence on Species Phylogeny section). Benchmarking gene trees ultimately compares two estimations: the estimated gene tree and the estimated species tree, and thus such a comparison has limitations and should be interpreted with caution.

To benchmark HOGs meaningfully, the field needs metrics that evaluate features unique to hierarchical orthology: consistency across taxonomic levels, correct placement of duplication events, and accurate reconstruction of ancestral genome content. Several strategies could help address the lack of HOG-specific benchmarks:

### Reference Gene Families with Curated Evolutionary Histories

Manually curated gene trees provide valuable benchmarks for evaluating HOG inference. Resources like SwissTree (Boeckmann et al. 2017), TreeFam-A (Ruan et al. 2008), and the reference orthologous groups (RefOGs) from Orthobench (Trachana et al. 2011) offer gold-standard datasets with well-characterized duplication and speciation histories. SwissTrees are curated in a large set of species, but only include 19 reference trees. TreeFam-A contains 1314 reference trees, but these gene families are restricted to 25 metazoan genomes and four plant and fungal outgroups (Ruan et al. 2008). Trachana et al. (2011) manually curated a set of 70 RefOGs with a focus on genes present in the last common ancestor or Bilateria. Emms and Kelly (2020) later updated the dataset using improved methods in phylogenetic tree inference and revised 31 of the 70 RefOGs. A potential limitation of these resources is that they are defined at a single level, making it easier for methods to overfit parameters to a specific taxonomic level. Expanding reference datasets to include orthologous groups defined at other speciation nodes would improve their utility for benchmarking hierarchical inference across broader taxonomic ranges.

Using these RefOG sets, the LEMOrtho platform provides a benchmarking framework based on the revised RefOGs (Kuznetsov et al. 2023). LEMOrtho assesses performance using multiple metrics, including precision, recall, F1 score, and the Variation of Information (VI) metric (Meilă, 2003) to quantify clustering similarity. Other relevant benchmarking measures include the number of fusions (where multiple RefOGs are incorrectly merged), splits (where a single RefOG is fragmented), and complex mismatches (substantial deviations from the RefOG structure) (Kriventseva et al. 2015; Kuznetsov et al. 2023). Notably, Kuznetsov et al. found that across methods, the majority of the RefOGs had an average F1 score over 80%, indicating generally high accuracy across methods (Kuznetsov et al. 2023). Differences nevertheless emerged: The number of RefOGs with F1 ≥ 85% was highest for SonicParanoid (62 out of 70), followed by OrthoDB’s OrthoLoger (61), OrthoFinder (58), OrthoMCL (56), and OMA (56). OMA ranked highest in precision (65 RefOGs ≥ 85%), while OrthoFinder had the best recall (64 RefOGs ≥ 85%). OrthoLoger showed the highest number of exact matches to RefOGs (20). While OMA’s GETHOGs produced the most split RefOGs events, its highest overall precision reflects the common trade-off in orthology inference: maximizing precision can come at the cost of increased fragmentation.

### Simulated Datasets

Simulations allow full control over evolutionary histories and provide ground-truth annotations of duplications, losses, and orthologs across a species tree. Several tools allow for simulating genome evolution such as ALF (Dalquen et al. 2012) and SLiM (Messer 2013). They could be used to evaluate whether HOG methods accurately place duplication events and infer the origin of gene families.

### Reference-Free Benchmarking

References-free metrics assess internal consistency and biological plausibility of HOGs without requiring curated datasets. One approach is to compare HOG predictions across multiple methods. Tools/databases like MetaPhOrs or DIOPT assess consistency between ortholog pairs inferred from phylogenetic trees obtained from different sources (Chorostecki et al. 2020; Hu et al. 2011). One could envision a similar comparison of graph-based or hybrid HOG inference methods. The Variation of Information metric implemented in the LEMOrtho benchmarking framework could be used to quantify the agreement between HOG predictions from different methods (Kuznetsov et al. 2023).

Additional properties of the HOGs themselves can be informative. The completeness score, as defined in (Altenhoff et al. 2024a, b), measures how many species are represented within a HOG, providing a rough indicator of whether a group is biologically plausible or artificially sparse based on the parsimony principle of implied losses. HOG size (the number of genes per HOG) is also a useful metric: an excess of very small HOGs may indicate over-splitting, while extremely large HOGs may result from overly permissive clustering.

Finally, the total number of HOGs at a given taxonomic level should approximate the expected size of the ancestral gene repertoire, with drastic deviations suggesting systematic biases.

## Conclusions and Future Perspectives

Hierarchical orthologous groups have become a powerful framework in comparative genomics. They provide an evolutionarily grounded representation of gene families across taxonomic levels, supporting a wide range of downstream analyses including phylogenomic profiling, ancestral genome reconstruction, gene family evolution, and functional annotation. The adoption of HOGs across orthology databases and methods highlights their utility, particularly as the number of available genomes continues to grow.

While the definition of a HOG is conceptually straightforward—homologs descending from a common ancestral gene at a given taxonomic level—extracting biologically relevant information from HOGs remains challenging for many users. This is compounded by fragmented software ecosystems, limited parsing and visualization options, and lack of standard outputs such as reconciled gene trees, ortholog/paralog lists, or phylogenetic profiles. There is a clear need for more interpretable, user-friendly platforms that expose the full richness of HOGs. Most current inference methods now blend graph-based and gene tree-based strategies, reflecting a broader shift toward hybrid approaches. However, the field still lacks robust benchmarking tailored to HOGs, making it difficult to assess or compare methods rigorously. Given the growing scale and complexity of genomic data, such benchmarks, along with confidence scores and improved support for domain-level orthology, will be essential.

Despite the challenges, the potential of HOGs remains high. They enable systematic insights into gene function, duplication histories, and ancestral genome content and can even support stable gene naming schemes. Future directions may include extending HOGs to phylogenetic networks to better accommodate non-vertical events like hybridization and lateral gene transfer. Ultimately, the accuracy and utility of HOGs will remain closely tied to the quality of gene annotations, though future methods may increasingly account for incomplete or fragmented data. Nevertheless, HOGs remain one of the few frameworks capable of integrating gene evolution at both deep and recent evolutionary timescales. Their hierarchical structure makes them uniquely suited for comparative questions that require resolution across diverse lineages. With ongoing efforts to scale, benchmark, and improve interpretability, HOGs are poised to remain central to evolutionary and functional genomics in the era of high-throughput comparative analysis.

## Data Availability

The supplemental code (Jupyter notebook) and data used to generate the plot in Fig. 7 are available at https://github.com/DessimozLab/ancestral_genomes.

## Abbreviations

*AGORA*: Algorithm for Gene Order Reconstruction in Ancestors
*CARs*: Contiguous Ancestral Regions
*GO*: Gene Ontology
*GSTD*: Generalized Species Tree Discordance
*HGT*: Horizontal Gene Transfer
*HOGs*: Hierarchical Orthologous Groups
*LCA*: Last Common Ancestor
*LECA*: Last Eukaryotic Common Ancestor
*LUCA*: Last Universal Common Ancestor
*MCL*: Markov clustering
*PAINT*: Phylogenetic Annotation and INference Tool
*QfO*: Quest for Orthologs
*RBH*: Reciprocal Best Hit
*RefOGs*: Reference Orthologous Groups
*RF*: Robinson–Foulds
*STAG*: Species Tree inference from All Genes
*STRIDE*: Species Tree Root Inference from gene Duplication Events
*VI*: Variation of Information
