# Correction: Reconstructing Evolutionary Histories with Hierarchical Orthologous Groups

**DOI:** 10.1007/s00239-025-10298-w

**Published:** 2025-12-27

**Authors:** Garance Sarton-Lohéac, Nikolai Romashchenko, Clément Marie Train, Sina Majidian, Natasha Glover

**Affiliations:** 1 Department of Fundamental Microbiology, University of Lausanne, 1015 Lausanne, Switzerland; 2 Department of Computational Biology, University of Lausanne, 1015 Lausanne, Switzerland; 3 SIB Swiss Institute of Bioinformatics, 1015 Lausanne, Switzerland; 4 Department of Computer Science, Johns Hopkins University, 3400, North Charles St., Baltimore, MD 21218 USA

**Correction to: Journal of Molecular Evolution (2025)** 10.1007/s00239-025-10277-1

In Table 1 of this article, in the first column “Method (algorithm)” the below words were incorrectly written. For completeness and transparency, the old incorrect and the corrected versions of Table 1 are displayed below.

In Row 1, the word “OrthoL-oger” was incorrect and should have read “Ortho-Loger”

In Row 5, the word “Gen-eTrees” was incorrect and should have read “Gene-Trees”

In Row 6, the word “USE-ARCH” was incorrect and should have read “U-SEARCH”

In Addition to the above in Figure 8 legend, the word Orth-oXML was incorrect and should have read “Ortho-XML”.

**Incorrect Table 1**

**Table:** 

Method (algorithm)	Method type	Similarity search method	Clustering algorithm	Alignment method (for tree building)	Tree reconstruction method	Reconciliation method	Database name	References
OrthoDB (OrthoL-oger)	graph-based	MMSeqs2	Triangulating and clustering all RBHs and in-paralogs	N/A	N/A	N/A	OrthoDB https://www.orthodb.org	(Kriventseva et al. 2008, 2015; Kuznetsov et al. 2023; Tegenfeldt et al. 2024; Zdobnov et al. 2021)
OMA (GETHOGs 2)	graph-based	Smith-Waterman	GETHOGS (identifies connected components + MinCut)	N/A	N/A	N/A	Omabrowser https://omabrowser.org	(Altenhoff et al. 2013; Altenhoff et al. 2024a, b; Roth et al. 2008; C.-M. Train et al. 2017)
PhylomeDB V5	gene tree-based	BLAST	seed gene + BLAST to other genomes	MUSCLE, MAFFT, KALIGN, M-Coffee	Maximum likelihood IQ-TREE with different models. Best model selected with BIC	Species overlap	PhylomeDB https://phylomedb.org	(Fuentes et al. 2022; Huerta-Cepas et al. 2014)
PANTHER	gene tree-based	Mapping sequences to curated families using HMMs	Tribe-MCL	MAFFT	GIGA	GIGA	PANTHER https://pantherdb.org	(Mi et al. 2007, 2013; , 2017; Thomas 2010; Thomas et al. 2022)
Ensembl- Compara (Gen-eTrees)	gene tree-based	BLAST + Smith-Waterman	hcluster_sg for phylogeny-aware clustering	M-Coffee or MAFFT for large groups	TreeBeST	TreeBeST	EnsemblCompara https://www.ensembl.org/info/genome/compara	(Herrero et al. 2016; Vilella et al. 2009)
Hieranoid 2	hybrid	BLAST, USE-ARCH possible	InParanoid	MUSCLE	Neighbor-Joining tree with Belvu	ETE3	HieranoidDB https://hieranoidb.sbc.su.se	(Kaduk & Sonnhammer 2017; Remm et al. 2001; Sonnhammer & Östlund 2015)
OMA (FastOMA)	hybrid	Mapping sequences to HOGs with OMAmer; linclust for unmapped seqs	GETHOGs 2	MAFFT	FastTree	Species overlap	N/A	(Majidian et al. 2025)
eggNOG 6.0	hybrid	Mapping sequences COGs with eggNOG mapper; SIMAP on Smith-Waterman RBHs for unmapped seqs	(COG) triangulating and clustering all RBHs	MUSCLE, M-Coffee, Clustal Omega, MAFFT	FastTree, PhyML	ETE3	eggNOG http://eggnog6.embl.de	(Hernández-Plaza et al. 2023; Huerta-Cepas et al. 2016a, b; Powell et al. 2012, 2014)
OrthoFinder 2.0	hybrid	BLAST	MCL	MAFFT	DendroBLAST + STAG + STRIDE	Species overlap + duplication-loss- coalescent model	N/A	(Emms & Kelly 2015, 2019)
MBGD	hybrid	DIAMOND for pangenome construction; BLASTP + SW for top-level ortholog grouping	DomClust + DomRefine (UPGMA domain-aware clustering)	FAMSA	FastTree	DomRefine	MBGD https://mbgd.nibb.ac.jp	(Uchiyama 2006; Uchiyama et al. 2025)

**Corrected Table** **1**

**Table 1:** 

Method (algorithm)	Method type	Similarity search method	Clustering algorithm	Alignment method (for tree building)	Tree reconstruction method	Reconciliation method	Database name	References
OrthoDB (Ortho-Loger)	graph-based	MMSeqs2	Triangulating and clustering all RBHs and in-paralogs	N/A	N/A	N/A	OrthoDB https://www.orthodb.org	(Kriventseva et al. 2008, 2015; Kuznetsov et al. 2023; Tegenfeldt et al. 2024; Zdobnov et al. 2021)
OMA (GETHOGs 2)	graph-based	Smith-Waterman	GETHOGS (identifies connected components + MinCut)	N/A	N/A	N/A	Omabrowser https://omabrowser.org	(Altenhoff et al. 2013; Altenhoff et al. 2024a, b; Roth et al. 2008; C.-M. Train et al. 2017)
PhylomeDB V5	gene tree-based	BLAST	seed gene + BLAST to other genomes	MUSCLE, MAFFT, KALIGN, M-Coffee	Maximum likelihood IQ-TREE with different models. Best model selected with BIC	Species overlap	PhylomeDB https://phylomedb.org	(Fuentes et al. 2022; Huerta-Cepas et al. 2014)
PANTHER	gene tree-based	Mapping sequences to curated families using HMMs	Tribe-MCL	MAFFT	GIGA	GIGA	PANTHER https://pantherdb.org	(Mi et al. 2007, 2013; , 2017; Thomas 2010; Thomas et al. 2022)
Ensembl- Compara (Gene-Trees)	gene tree-based	BLAST + Smith-Waterman	hcluster_sg for phylogeny-aware clustering	M-Coffee or MAFFT for large groups	TreeBeST	TreeBeST	EnsemblCompara https://www.ensembl.org/info/genome/compara	(Herrero et al. 2016; Vilella et al. 2009)
Hieranoid 2	hybrid	BLAST, U-SEARCH possible	InParanoid	MUSCLE	Neighbor-Joining tree with Belvu	ETE3	HieranoidDB https://hieranoidb.sbc.su.se	(Kaduk & Sonnhammer 2017; Remm et al. 2001; Sonnhammer & Östlund 2015)
OMA (FastOMA)	hybrid	Mapping sequences to HOGs with OMAmer; linclust for unmapped seqs	GETHOGs 2	MAFFT	FastTree	Species overlap	N/A	(Majidian et al. 2025)
eggNOG 6.0	hybrid	Mapping sequences COGs with eggNOG mapper; SIMAP on Smith-Waterman RBHs for unmapped seqs	(COG) triangulating and clustering all RBHs	MUSCLE, M-Coffee, Clustal Omega, MAFFT	FastTree, PhyML	ETE3	eggNOG http://eggnog6.embl.de	(Hernández-Plaza et al. 2023; Huerta-Cepas et al. 2016a, b; Powell et al. 2012, 2014)
OrthoFinder 2.0	hybrid	BLAST	MCL	MAFFT	DendroBLAST + STAG + STRIDE	Species overlap + duplication-loss- coalescent model	N/A	(Emms & Kelly 2015, 2019)
MBGD	hybrid	DIAMOND for pangenome construction; BLASTP + SW for top-level ortholog grouping	DomClust + DomRefine (UPGMA domain-aware clustering)	FAMSA	FastTree	DomRefine	MBGD https://mbgd.nibb.ac.jp	(Uchiyama 2006; Uchiyama et al. 2025)

The original article has been corrected.

